# High-accuracy prediction of colorectal cancer chemotherapy efficacy using machine learning applied to gene expression data

**DOI:** 10.3389/fphys.2023.1272206

**Published:** 2024-01-18

**Authors:** Soukaina Amniouel, Mohsin Saleet Jafri

**Affiliations:** ^1^ School of Systems Biology, George Mason University, Fairfax, VA, United States; ^2^ Center for Biomedical Engineering and Technology, University of Maryland School of Medicine, Baltimore, MD, United States

**Keywords:** colorectal cancer, FOLFOX, FOLFIRI, chemoresistance, machine learning, gene expression, feature selection

## Abstract

**Introduction:** FOLFOX and FOLFIRI chemotherapy are considered standard first-line treatment options for colorectal cancer (CRC). However, the criteria for selecting the appropriate treatments have not been thoroughly analyzed.

**Methods:** A newly developed machine learning model was applied on several gene expression data from the public repository GEO database to identify molecular signatures predictive of efficacy of 5-FU based combination chemotherapy (FOLFOX and FOLFIRI) in patients with CRC. The model was trained using 5-fold cross validation and multiple feature selection methods including LASSO and VarSelRF methods. Random Forest and support vector machine classifiers were applied to evaluate the performance of the models.

**Results and Discussion:** For the CRC GEO dataset samples from patients who received either FOLFOX or FOLFIRI, validation and test sets were >90% correctly classified (accuracy), with specificity and sensitivity ranging between 85%-95%. In the datasets used from the GEO database, 28.6% of patients who failed the treatment therapy they received are predicted to benefit from the alternative treatment. Analysis of the gene signature suggests the mechanistic difference between colorectal cancers that respond and those that do not respond to FOLFOX and FOLFIRI. Application of this machine learning approach could lead to improvements in treatment outcomes for patients with CRC and other cancers after additional appropriate clinical validation.

## 1 Introduction

Colorectal cancer (CRC) is the most frequent malignant disease of the gastrointestinal tract, the third most frequent cancer affecting both men and women and is one of the leading causes of cancer-related morbidity and mortality in spite of widespread, effective measures of preventive screening, and major advances in treatment options (Fouad et al., 2018; Sung et al., 2021). In recent decades, the overall long-term outcome of patients curatively resected has not significantly changed. The 5-year survival rate for CRC is 63% but drops to 14% for metastatic CRC. More than half of colorectal adenocarcinomas are still diagnosed only when the disease involves regional or distant structures (Araghi et al., 2021). Thus, further investigation is still needed to develop effective approaches for medical intervention.

Chemotherapy remains one of the most used therapeutic options for CRC patients, and is usually combined with surgery, radiotherapy, immunotherapy, and targeted molecular therapy (Salonga et al., 2000; Showalter et al., 2008; Zhang et al., 2020b). Advances in CRC treatment have led to the development of two combinations of cytotoxic drugs, FOLFIRI (FOL = Leucovorin Calcium (Folinic Acid), F = Fluorouracil and IRI = Irinotecan Hydrochloride) and FOLFOX (FOL = Leucovorin Calcium (Folinic Acid), F = Fluorouracil and OX = Oxaliplatin) (Douillard et al., 2000; Pelley, 2001). These drugs have been used as initial intensive therapy for metastatic CRC in patients with good tolerance. Oxaliplatin and irinotecan agents have been proven to have efficacy in the treatment of CRC. Irinotecan inactivates topoisomerase I via its active metabolite SN38 and arrests cell division (Bailly, 2019). Oxaliplatin, on the other hand, acts primarily by causing inter- and intra-strand cross-links in DNA, thereby inhibiting DNA synthesis and triggering apoptosis (Wiseman et al., 1999; Alcindor and Beauger, 2011). The overall survival of advanced colorectal cancer patients has been improved thanks to the availability of these chemotherapy regimens.

In spite of advances in cytotoxic therapy, resistance to chemotherapy remains one of the greatest challenges in long-term management of incurable metastatic disease and eventually contributes to death as cancer find ways to become tolerant of pharmaceutical treatments (Dallas et al., 2009; Li et al., 2017; Mansoori et al., 2017). Studies on predictive biomarkers useful for differentiating between which cytotoxic agent, FOLFOX or FOLFIRI, should be used to treat patients are currently lacking. In stage III metastatic CRC, patients responded to FOLFOX and FOLFIRI with a 54% and 56% response rate (Tournigand et al., 2004). In another study in patients with advanced CRC, patients responded to FOLFOX and FOLFIRI with a 34% and 31% response rate, respectively (Colucci et al., 2005). Given the similar patient response rates in these studies, the criteria for selecting an optimal drug choice for a given patient remains unclear. Therefore, a meta-study based on predictive gene signatures for FOLFOX and FOLFIRI is now highly desirable in a cohort of patients treated with these regimens.

Recent advances in the ability to generate molecular data, as well as parallel advances in the fields of artificial intelligence, specifically machine learning (ML) (de Jong et al., 2021), have led to remarkable opportunities to understand these resistance mechanisms and develop personalized treatment strategies to overcome resistance (Perez-Gracia et al., 2017; Frohlich et al., 2018). Numerous studies have already been conducted for predicting drug-response in other cancer types such as breast cancer (Del Rio et al., 2007). However, there is lack of studies on the possible added value of this approach for predicting drug response in CRC (Del Rio et al., 2007). Thus, the aim of this study is to build machine learning models for predicting the response to FOLFOX and FOLFIRI treatment in patients with CRC using gene expression profiles of primary and metastatic colon cancer tissues.

## 2 Materials and methods

### 2.1 Data

In this study, the raw data (CEL-files) of the colon cancer gene expression datasets was retrieved from the public functional genomics data repository NCBI-GEO database (http://www.ncbi.nlm.nih.gov/geo/last accessed on 17 September 2021), using the getGEO function implemented in the R library GEOquery (Davis and Meltzer, 2007). Affy package in R was used to transform the CEL files of the tumor samples into an expression matrix (Gautier et al., 2004). “Colon-Cancer,” “Chemotherapy,” “Expression profiling by array,” and “Homo-sapiens” were used as keywords to query all the experimental studies that have probed the gene expression profile within colon tumors of patients who are responders to the drug against those who are not responders. The chemotherapy regimens of interest FOLFOX and FOLFIRI. This approach yielded five different studies, from which the samples of two chemotherapy types (FOLFOX and FOLFIRI) were separated and grouped accordingly. Table 1 presents the summary of the expression datasets that are included in this study.

**TABLE 1:** Description of each dataset for two different Chemotherapy regimens. GPL96 = Affymetrix GeneChip Human Genome U133 Array (HG-U133A); GPL570 = Affymetrix GeneChip Human Genome U133 Plus 2.0 Array (HG-U133Plus2).

GEO accession	Platform	No. of samples	Title/Description
GSE19860	GPL570	40 (15 responders +25 non-responders)	Prediction of response to FOLFOX
GSE72970	GPL570	124 (63 responders +61 non-responders)	Molecular subtypes of metastatic colorectal cancer are predictive of patient response to chemo and targeted therapies
GSE28702	GPL570	83 (42 responders +41 non-responders)	CRC samples for FOLFOX therapy prediction
GSE62080	GPL570	21 (9 responders +12 non-responders)	Gene expression signature in advanced colorectal cancer patients select drugs and response for the use of leucovorin, fluorouracil, and irinotecan
GSE62321	GPL96	57 (26 responders +31 non-responders)	Specific extracellular matrix remodeling signature of colon hepatic metastases

### 2.2 Inclusion and exclusion criteria

The inclusion criteria in this study were set as follows: (1) patients with colorectal cancer; (2) patients who received FOLFOX or FOLFIRI chemotherapy regimen; (3) microarray expression profiling datasets; (4) sample size of at least 15 for each dataset; (5) available information about the drug response (i.e., responder to the drug vs. non-responder to the drug). Exclusion critieria were as follows: (1) datasets contain cell-line or xenograft samples; (2) samples who received preoperative bevacizumab therapy or other immunotherapy; (3) samples with missing information about the drug type; (4) samples with missing information about the drug response; (5) and samples who received a drug combination of FOLFOX and FOLFIRI such as FOLFOXIRI.

### 2.3 Machine learning framework

The machine learning framework used to predict the chemotherapy response includes the followings steps: data integration and pre-processing, data splitting using 5-fold cross validation, and feature selection.

#### 2.3.1 Data integration and pre-processing

The expression intensities for all genes across the samples were background corrected and normalized using the robust multiarray average (RMA) with the help of the probe sequence from the package gcrma, as implemented in the BiocManager software suite. To increase the sample size and improve the statistical significance of the results, a minimum of two gene expression datasets for each chemotherapy regimen were merged. Because each platform has a different set of protocols and studies, combining the expression datasets can result in discrepancies. As a result, the most effective approach was to merge the datasets produced by the same platform. Genes/probes with minor sample variance and low median expression levels were removed from RMA data using the nsFilter function of the “genefilter” package (version 1.60.0) in R. Then, t-tests were performed in the LIMMA package to identify differentially expressed genes (DEGs). The threshold value for DEGs was represented by a *p*-value <0.05 and |log_2_ fold change (FC)| ≥1. Each sample was then z-score normalized to represent the expression’s distribution. The feature selection was then applied to the pre-processed differential gene expression.

#### 2.3.2 Data splitting using 5-fold cross validation method

The machine learning model is initially fitted on a training data set. The model performance is then evaluated on the validation data set. Often when the data set is small a cross-validation procedure is used where the data is separated into a training and validation set in several iterations to train and validate the model. A test data set is a separate (independent) data set that has not been used at all in the training and validation of the model.

Using the function “create folds” available in the R package “caret”, samples were randomly split to the training and test set. The training set is split into 5 subsets of approximately equal size.

#### 2.3.3 Feature selection

In such large-scale machine learning applications, feature selection is a critical step in maximizing the benefits of big data while overcoming the associated challenges and costs. It enhances the machine learning application in a variety of ways, including faster computation speed with a smaller set of features, more accurate prediction by removing features and avoiding overfitting, and easier interpretation because only the most important feature set is included in the modeling process. There are numerous feature selection methods available for condensing the feature set. These methods can be loosely classified as filter methods, wrapper methods, and embedded methods. In this study, filter and embedded methods were applied to identify relevant variables associated with FOLFOX/FOLFIRI drug response.

### 2.4 Variable selection using LASSO and varSelRF

The variable selection using random forest (varSelRF) and Least Absolute Shrinkage and Selection Operator (LASSO) methods were employed to select the genes with the best predictive power. These methods were chosen not only because they return a small set of gene candidates that have high predictive power but also, they require a minimum fine-tuning of parameters as the default parameter values which often deliver the best performance.

The random forest variable selection (varSelRF) method uses regression trees for classification. Bootstrap samples are used to build the classification tree (Sharma and Dey, 2021). Every branch of the tree has a different set of candidate variables, and each branch’s candidate variables are chosen at random. Bootstrap aggregation (bagging) and feature selection are combined in this way to generate trees in RF. To obtain low-bias trees, each tree is developed entirely, and then bagging and random selection of variables is performed to facilitate low correlation of the various trees. The ntree parameter was set to its default value of 2000 and the mtry parameter was set to its default value (Diaz-Uriarte, 2007).

LASSO is a type of regularization regression method to fit a generalized linear model. Based on the concept of penalizing the regression model (L1-norm), LASSO squashes the regression coefficient for the least-contributing variable to zero (Sharma and Dey, 2021). LASSO performance excels when the data is high-dimensional and low-sample, and when only a few variables have large coefficients. Numerous research has shown that LASSO is a promising feature selection model (Hua, 2020; Ghosh Roy et al., 2021).

Using the outcomes obtained, the regression coefficients were utilized to create a scoring system that assigns weights to the selected signature. The formula employed for this purpose is as follows:

$$Prediction\;Score=\sum_{i=0}^{n}\left(\;\beta_{i}\times x_{i}\right)$$ (1)

In Equation 1, “n” denotes the sample size, while “β” represents the regression coefficient associated with the selected signature (Fu et al., 2021). The regression coefficient is obtained through LASSO logistic regression. Additionally, “x” signifies the expression value corresponding to the selected signature (Fu et al., 2021).

### 2.5 Machine learning algorithms for classification

The R packages RandomForest and e1071 were used to train two different machine learning algorithms: a random forest and a support vector machine (SVM). To compare the efficacy of the models, the following metrics were measured:

$$Accuracy=\frac{\left(TP+TN\right)}{\left(TN+FN+FP+TP\right)}$$ (2)

$$Sensitivity=\frac{TP}{\left(TP+FN\right)}$$ (3)

$$Specificity=\frac{TN}{\left(TN+FN\right)}$$ (4)

In Equations 2–4, the TP, TN, FN, and FP represent true positive, true negative, false negative, and false positive predictions respectively made by classification model for each chemotherapy regimen response (responders (R) denoted positive and non-responders (NR) is denoted negative). For further comparative analysis, the receiver operating characteristics (ROC) curve was plotted and compared to the area under the curve (AUC) obtained by the best models. Finally, the best machine learning model, fine-tuned to predict FOLFOX and FOLFIRI drug response, was applied to the test set. These methods were implemented using R language programming version 4.0.1. On an Intel Core-i9 CPU with 16 GB of RAM, and 64-bit Windows 10 configuration.

### 2.6 Functional enrichment analysis

To investigate the association between the predictors of our model and cellular function, a functional enrichment analysis was conducted using the web tool NetworkAnalyst (https://www.networkanalyst.ca/last accessed on 15 January 2023) (Zhou et al., 2019). NetworkAnalyst web-interface was used to visualize the interactions among the gene products based on the protein-protein interaction (PPI) data in the International Molecular Exchange Consortium (IMEx) database using the default parameters and first-order network (Shoily et al., 2021). IMEx is a curated database containing non-redundant set of interaction data from a broad taxonomic range of organism (Orchard et al., 2012; Shoily et al., 2021). The gene ontology (GO) categories including biological process (BP), molecular function (MF), and cellular component (CC) with false discovery rate (FDR) ≤ 0.05 were identified from the gene ontology database based on the PPI networks derived through IMEx. The pathways that incorporate these gene products (with false discovery rate (FDR) ≤ 0.05) were retrieved from the Kyoto Encyclopedia of Genes and Genomes (KEGG) pathway database based on the PPI networks derived through IMEx (Kanehisa et al., 2017; Shoily et al., 2021).

### 2.7 Biological pathway analysis

The canonical pathway enriched by differential genes was performed using Ingenuity Pathway Analysis (IPA). IPA is a web-based software application (Ingenuity Systems http://www.ingenuity.com) that identifies biological pathways and functions relevant to biomolecules of interest (He et al., 2021). A core analysis was first constructed, and then a list of differential genes with their probe identification, FDR value and logarithmic fold change were uploaded to IPA (He et al., 2021). Enrichment pathways of differential genes were generated based on the Ingenuity Pathway Knowledge Data Base.

## 3 Results

**FIGURE 1:**
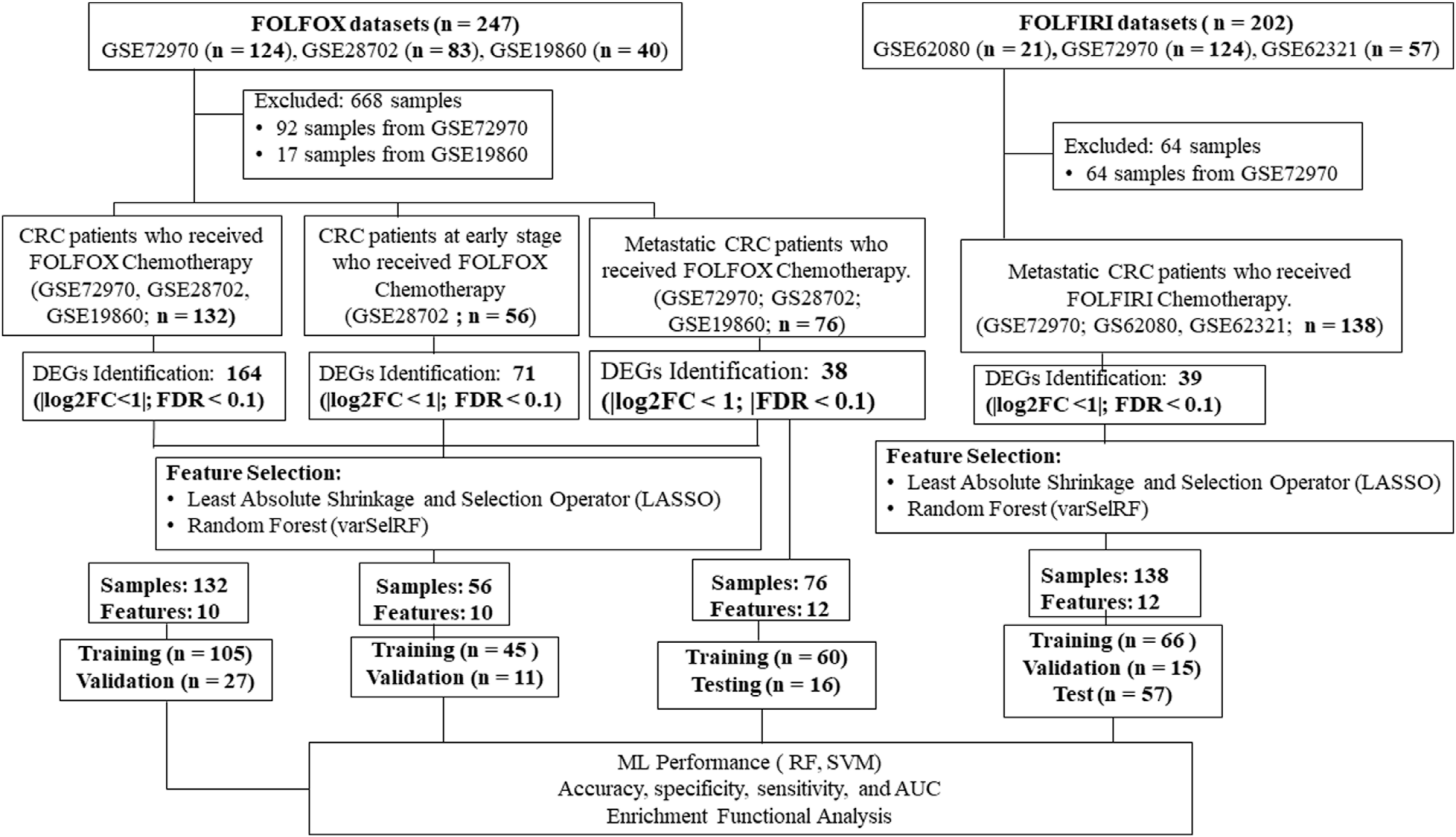
A multi-stage analysis methodology is applied in this study. Gene expression profiling datasets of human colorectal tissues were collected from the NCBI-GEO database. The datasets were analyzed using the robust multi-array average method in R to identify differentially expressed genes (DEGs). Feature selection methods were performed using LASSO and varSelRF methods to identify gene signature related to each chemotherapy drug (i.e., FOLFOX or FOLFIRI). The performance of the machine learning models was evaluated using random forest and support vector machine algorithms. Functional enrichment analysis of the gene signatures was performed to identify significantly enriched pathways and Gene Ontology (GO) terms. Protein-protein interaction networks were reconstructed around the gene signatures.

Our goal was to use tumor gene expression profiles to predict patients’ response to drugs. An overview of our framework is shown in Figure 1. The details are included in the section of materials and methods. A series of meta-analyses were performed to develop a machine learning model and identify biomarkers to predict the following: 1) FOLFOX responders vs. non-responders in all stages of CRC, 2) FOLFOX responders vs. non-responders at early stages of CRC, 3) FOLFOX responders vs. non-responders among patients with metastatic CRC, 4) responders vs. non-responders in samples who received FOLFIRI chemotherapy, 5) machine learning model application to predict effectiveness of alternate chemotherapy regimen All datasets in this study are identified by unique GEO accession numbers which are provided in the material and methods section. Each GEO submission file includes a brief overview of the experimental paradigm as well as a link to the published report, if available.

### 3.1 FOLFOX responders vs. non-responders of CRC

The first analysis of colorectal cancer patients identified significant genes separating FOLFOX responders from non-responders. In this step, the stage of the disease was not a significant factor. This part of analysis was conducted to compare genes found in this study to those identified in previous studies.

The GSE28702, GSE19860, and GSE72970 datasets which were generated by the Affymetrix microarray GPL570 platform, were combined to obtain a total of 67 non-responders and 65 responders of CRC patients treated with FOLFOX chemotherapy. The samples who received FOLFIRI drug were removed from GSE72970 dataset before the start of the analysis. The cross-validation method split the combined dataset into a training set consisting of a total of 105 (53 non-responders and 52 responders) samples and validation set consisting of a total of 27 (14 non-responders and 13 responders) samples.

After integrated bioinformatics analysis, a total of 164 differentially expressed genes (DEGs) between pre-chemotherapy tissue samples of non-responders and responders of CRC patients treated with FOLFOX were identified including 142 upregulated genes and 22 downregulated genes.

**FIGURE 2:**
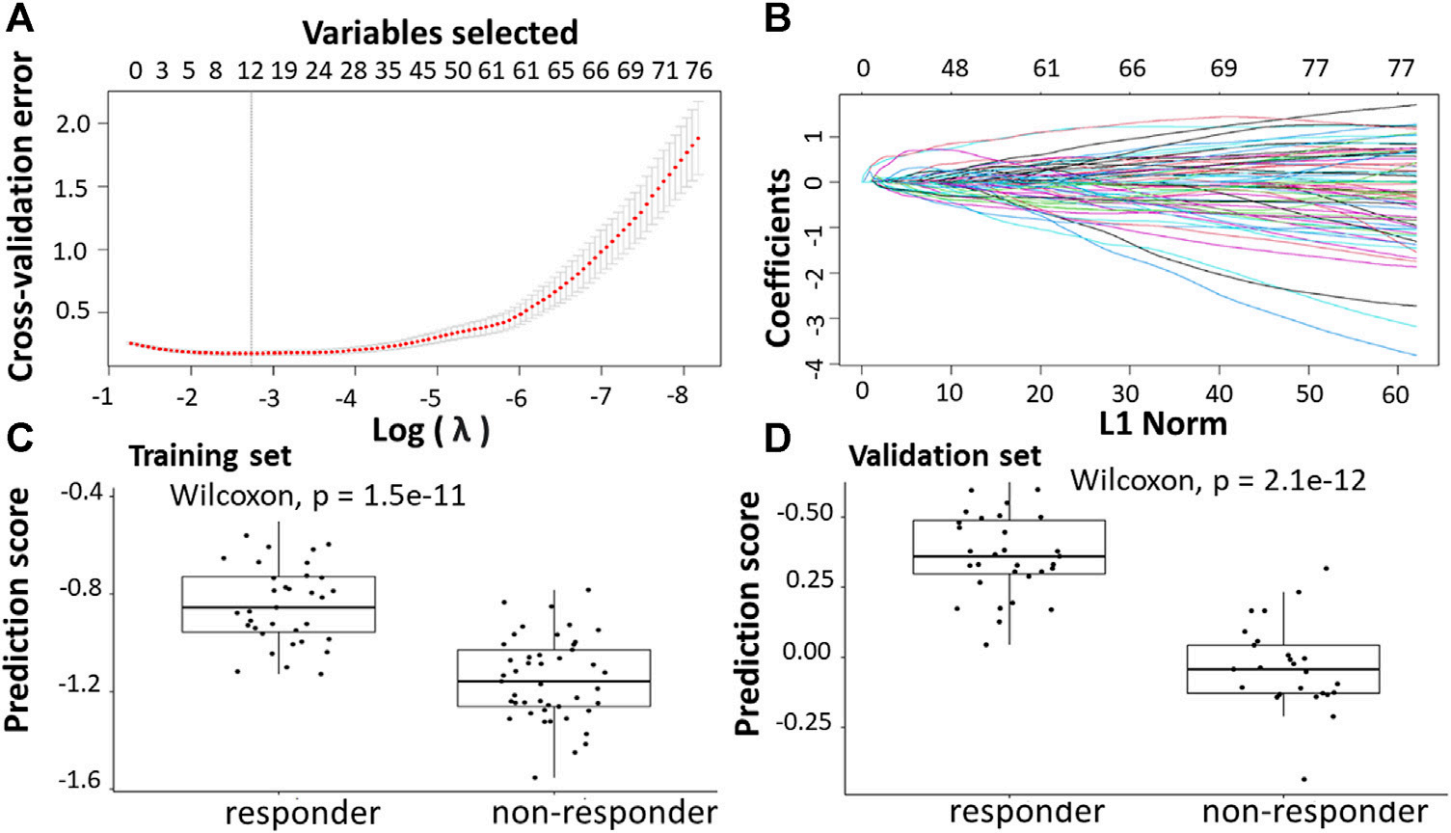
Construction of LASSO model for patients will all stages of CRC who received FOLFOX therapy. **(A)** Ten-fold cross-validation for tuning parameter selection in the LASSO model. **(B)** LASSO coefficient profiles of the training set. **(C)** The prediction score of the classifier (Equation 5) was higher in responder than in non-responder samples in the training set. **(D)** The prediction score of the classifier was higher in responder than non-responder samples in the validation set.

Following the identification of DEGs, the feature selection methods, LASSO and varSelRF, were applied to extract informative genes that have maximum relevance among DEGs. LASSO method identified 12 genes that considered to be relative to the drug response prediction. These genes were identified by selecting the optimal λ that was identified by performing the ten-fold cross-validations. The value of λ was determined by the minimum cross-validation error and was denoted as λmin. In this case, the λmin was equal to 0.0651, resulting in 12 non-zero coefficients including *CFAP92, DCDC2B, LTA4H, AP5Z1, LRRC3, SH3GLB1, CARM1, TRIM3, PPDPF, GPN3, GTF2A1, HELZ2* (Figures 2A,B). The expression of these genes was then used to evaluate the prediction score generated by the identified 12-genes that differentiate between the group of responders and non-responders. The following formula was used to calculate the prediction score of the identified genes:

$$\begin{array}{ll} Prediction\;Score & =\text{CFAP}92\times \left(0.0707023790552402\right)+\text{LTA}4H\times \left(-0.0351545549822743\right)+\text{SH}3\text{GLB}1\times \left(-0.0408850596289773\right)\\ & +\text{CARM}1\times 0.383628162287505+\text{PPDPF}\times 0.080928167572473+\text{GPN}3\times 0.0586206766352588\\ & +\text{TRIM}3\times 0.161462309025774+\text{HELZ}2\times 0.01687976195595+\text{LRRC}3\times 0.438014842725689\\ & +\text{LOC}100652999\times 0.14063710120844+\text{GTF}2A1\times 0.0776912702849236+\text{AP}5Z1\times \left(-0.022687801677234\right) \end{array}$$ (5)

The results showed that these identified genes were able to differentiate between the group of responders and non-responders. As shown in the figure, the responders have higher prediction scores compared to the non-responders. This was also elucidated in the plot that represents the validation set (Figures 2C,D).

In the meantime, the varSelRF method identified 11 genes including, *PIDD1, CFAP92, LTA4H, AP5Z1, SH3GLB1, CARM1, TRIM3, PPDPF, GPN3, GTF2A1, HELZ2.* Using these methods, the genes were continuously evaluated. The gene set with best prediction performance was used for further analysis. Ten genes were identified as relevant genes from both methods, including Cilia And Flagella Associated Protein 92 (***CFAP92***), Leukotriene A4 Hydrolase (***LTA4H***), SH3 Domain Containing GRB2 Like, Endophilin B1 (***SH3GLB1***), Adaptor Related Protein Complex 5 Subunit Zeta 1 (***AP5Z1***), Coactivator Associated Arginine Methyltransferase 1 (***CARM1***), Tripartite Motif Containing 3 (***TRIM3***), Pancreatic Progenitor Cell Differentiation And Proliferation Factor (***PPDPF***), GPN-Loop GTPase 3 (***GPN3***), General Transcription Factor IIA Subunit 1 (***GTF2A1***), and Helicase With Zinc Finger 2 (***HELZ2***). From the differential expressed genes analysis, the genes *CFAP92, AP5Z1, CARM1, TRIM3, PPDPF* were downregulated and the *LTA4H, SH3GLB1, GPN3, GTF2A1, HELZ2* genes were upregulated.

The assessment of model performance was performed in training and validation sets according to accuracy, sensitivity, specificity, and AUC. As shown in Table 2, the top machine learning algorithm was random forest, though there was no significant difference between random forest and SVM algorithm.

**TABLE 2:** Comparison of different classification methods on training and validation sets using the combination of LASSO and varSelRF method.

Model		FOLFOX (LASSO & VarSelRF)	
		Random forest (RF)	Support vector machine (SVM)
Training (n = 105)	Accuracy	1	0.92
	95% CI	(0.95, 1)	(0.77, 0.95)
	Sensitivity	1	0.92
	Specificity	1	0.84
Validation (n = 27)	Accuracy	1	0.96
	95% CI	(0.95, 1)	(0.75, 1)
	Sensitivity	1	0.92
	Specificity	1	1
	AUC	1	0.96

For the training set, random forest algorithm achieved an accuracy of 1 with 95% CI ranging between 0.95 and 1. The sensitivity and specificity were equal to 1. Support vector machine, on the other hand, achieved an accuracy of 0.95 with 95% CI ranging between 0.77 and 0.95. The sensitivity and specificity are equal to 0.92 and 0.84 respectively (Table 2).

For the validation set, random forest algorithm had an accuracy of 1 with 95% CI ranging between 0.95 and 1. The sensitivity, specificity, and area under curve (AUC) are equal to 1. The support vector machine algorithm achieved an accuracy of 0.96 with 95% CI ranging between 0.75 and 1. The sensitivity, specificity, and AUC are equal to 0.92, 1, 0.96 respectively (Table 2).

The protein-protein interaction (PPI) networks generated through IMEx indicate (direct and indirect) interactions among these gene encoding proteins (Supplementary Figure S1). IMEx consortium annotates experimental interaction evidence directly from the source publications and provides curated non-redundant set of physical and molecular interaction data (Shoily et al., 2021). As shown in Supplementary Figure S1, the PPI network comprises 208 nodes (genes with connections to other genes) and 216 edges (connections between nodes) with 5 out of 10 genes being hub genes (genes with many connections to other genes). For instance, *CARM1*, *LTA4H*, *GTF2A1, TRIM3,* and *SH3GLB1* had the highest number of interactions with other genes. Based on the PPI network predicted using IMEx, the signature genes encoding proteins have no known direct functional effect on each other. *CARM1* connects to *LTA4H*, *TRIM3*, *SH3GLB1*, and *GPN3* via *ELVAL1*, *UBE2D4*, *CUL2*, and *CUL5* respectively. *CARM1* also connects to *GTF2A1* through *TERF2*, *CREB1*, and *HNRNPA1*. In addition, *LTA4H* connects to *GTF2A1* via *SIRT1*, whereas *GPN3* connects to *SH3GLB1* through *UBD* gene encoding protein. *TRIM3* and *CARM1* genes are connected via *UBE2D4*. *GPN3*, *SH3GLB1*, *AP5Z1*, *HELZ2*, *PPPDPF*, and *GTF2A1* interact directly with *UBC*.

### 3.2 FOLFOX responders vs. non-responders at early stages of CRC

Further subgroup analysis was carried out because some of the datasets had a combination of primary and metastatic lesions. In this analysis, only primary tumor samples were focused on identifying genes separating responders from non-responders in the early stages of cancer. The GSE28702 dataset derived from GPL570 consisted of 56 primary CRC samples from patients who had received first-line FOLFOX-based treatment. The metastasis samples from the dataset were excluded in this analysis. 45 (18 non-responders and 27 responders) samples from the datasets were used as a training set, while the remaining 11 (7 non-responders and 4 responders) samples were used as a validation set. Due to low sample size and skewness of individual gene expression levels in the training dataset, the bootstrap *t*-test was implemented to reduce the likelihood of false positives. A gene with an FDR≤ 0.05 and |log2FC| ≥1 was identified as differentially expressed gene (DEG).

After integrated bioinformatics analysis, 71 differentially expressed genes (DEGs) between pre-chemotherapy tissue samples of non-responders and responders of CRC patients treated with FOLFOX were identified including 55 upregulated genes and 16 downregulated genes.

**FIGURE 3:**
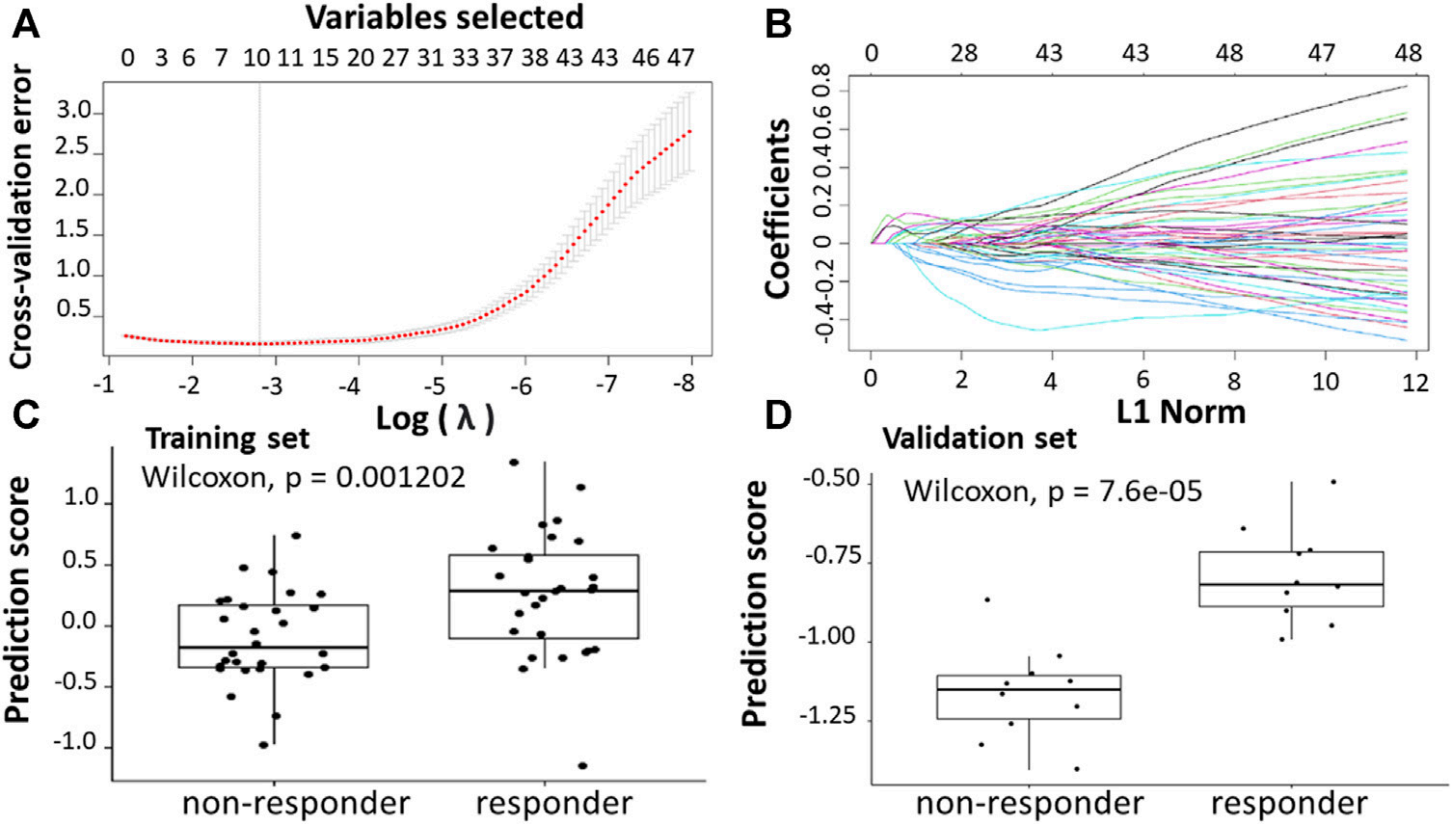
Construction of LASSO model. **(A)** Ten-fold cross-validation for tuning parameter selection in the LASSO model. **(B)** LASSO coefficient profiles of the training set. **(C)** The prediction score of the classifierclassifier (Equation 6) was higher in responder than in non-responder samples in the training set. **(D)** The prediction score of the classifier was higher in responder than non-responder samples in the validation set.

Following the identification of DEGs, the feature selection methods, LASSO and varSelRF, were applied to select gene signatures among DEGs. The LASSO method identified 10 genes that are relative to the drug response prediction. These genes were identified by selecting the optimal λ that was identified by performing the ten-fold cross-validations. The value of λ was determined by the minimum cross-validation error and was denoted as λmin. In this case, the λmin was equal to 0.0605, resulting in 10 non-zero coefficients including *FOXA1, KRT23, GRM8, HOXA11, HOXA10, ABCB1, LEFTY1, CHRM3, OLMF4, LYZ* (Figures 3A,B). The expression of these genes was then used to evaluate the prediction score generated by the identified 10-genes that differentiate between the group of responders and non-responders. The following formula was used to calculate the prediction score of the identified genes:

$$\begin{array}{ll} Prediction\;Score & =\text{FOXA}1\times 0.0252272039743314+\text{KRT}23\times 0.147656847485106+\text{GRM}8\times 0.0609620384129753\\ & +\text{HOXA}11\times \left(-0.0329157407927049\right)+\text{HOXA}10\times 0.0501485865470492+\text{ABCB}1\times 0.0545018052768938\\ & +\text{LEFTY}1\times 0.0802540599090828+\text{CHRM}3\times \left(-0.0342169746495821\right)+\text{OLMF}4\times 0.115227059404438\\ & +\text{LYZ}\times 0.0685852089429017 \end{array}$$ (6)

The results showed that these identified genes were able to differentiate between the group of responders and non-responders. As shown in the figure, the responders have higher prediction scores compared to the non-responders. This was also elucidated in the plot that represents the validation set (Figures 3C,D).

The varSelRF method also identified the same 10 genes. Using these methods, the genes were continuously evaluated and the gene set that received the best prediction performance was used for further analysis. Ten genes were identified as relevant genes from both methods, including Forkhead Box A1 (***FOXA1***), Keratin 23 (***KRT23***), (Glutamate Metabotropic Receptor 8 (***GRM8***), (Homeobox A11 (***HOXA11***), (Homeobox A10 (***HOXA10***), ATP Binding Cassette Subfamily B Member 1 (***ABCB1***), Left-Right Determination Factor 1 (***LEFTY1***), Cholinergic Receptor Muscarinic 3 (***CHRM3***), Olfactomedin 4 (***OLMF4***), and Lysozyme (***LYZ***).

The assessment of model performance was performed in training and validation sets according to accuracy, sensitivity, specificity, and AUC. As shown in Table 3, the top machine learning algorithm was random forest.

**TABLE 3:** Comparison of different classification methods on training and validation using the combination of LASSO and varSelRF method.

Model		FOLFOX (LASSO & VarSelRF)	
		Random forest (RF)	Support vector machine (SVM)
Training (n = 45)	Accuracy	1	0.86
	95% CI	(0.92, 1)	(0.73, 0.95)
	Sensitivity	1	0.77
	Specificity	1	0.92
Validation (n = 11)	Accuracy	0.99	0.90
	95% CI	(0.72, 1)	(0.59, 1)
	Sensitivity	0.99	0.86
	Specificity	0.98	1
	AUC	0.99	0.93

For the training set, random forest algorithm achieved an accuracy of 1 with 95% CI ranging between 0.92 and 1. The sensitivity and specificity were equal to 1. Support vector machine, on the other hand, achieved an accuracy of 0.86 with 95% CI ranging between 0.73 and 0.95. The sensitivity and specificity are equal to 0.77 and 0.92 respectively (Table 3).

For the validation set, random forest algorithm had an accuracy of 0.99 with 95% CI ranging between 0.72 and 1. The sensitivity, specificity, and area under curve (AUC) are equal to 0.99, 0.98, and 0.99 respectively. The support vector machine algorithm achieved an accuracy of 0.90 with 95% CI ranging between 0.59 and 1. The sensitivity, specificity, and AUC are equal to 0.86, 1, 0.93 respectively (Table 3).

The protein-protein interaction (PPI) networks generated through IMEx indicate interactions (direct and indirect) among the gene encoding proteins related with resistance to the FOLFOX regimen in patients with early-stage CRC (Supplementary Figure S2). As shown in Supplementary Figure S2, the PPI network comprises 114 nodes and 112 edges. 6 out of 10 genes formed hub genes. For instance, *HOXA10*, *HOXA11*, *ABCB1*, *FOXA1*, and *LYZ* had the highest number of protein interactions. Based on the PPI network predicted using IMEx, the signature genes have no known direct functional effect on each other. *HOXA10* connects to *HOXA11* via *ASXL1*, *EZH2*, and *HDAC2*. On the other hand, *HOXA10* connects to *ABCB1* via *EP300* and *ESR1*. Moreover, *LYZ* connects to *FOXA1* via the gene encoding protein *Jun*. In addition, *ABCB1*, *LEFTY1*, *KRT23*, *GRM8*, *LYZ*, *FOXA1*, and *HOXA11* interact directly with UBC.

### 3.3 FOLFOX responders vs. non-responders among patients with metastatic CRC

This analysis focused on selecting genes separating responders from non-responders in metastatic CRC patients. The GSE19860 and GSE72970 datasets were used in this step along with the metastatic samples from the GSE28702 dataset. These three datasets, which were generated by the GPL570 platform, were combined to yield a total of 42 non-responders and 34 responders of metastatic CRC patients with mFOLFOX chemotherapy. A gene with an FDR≤ 0.05 and |log2FC| ≥1 was identified as differentially expressed gene (DEG). Following datasets preprocessing, 39 differential expressed genes (DEGs) between pre-chemotherapy tissue samples of non-responders and responders of CRC patients treated with FOLFOX were identified including 18 upregulated genes and 20 downregulated genes.

**FIGURE 4:**
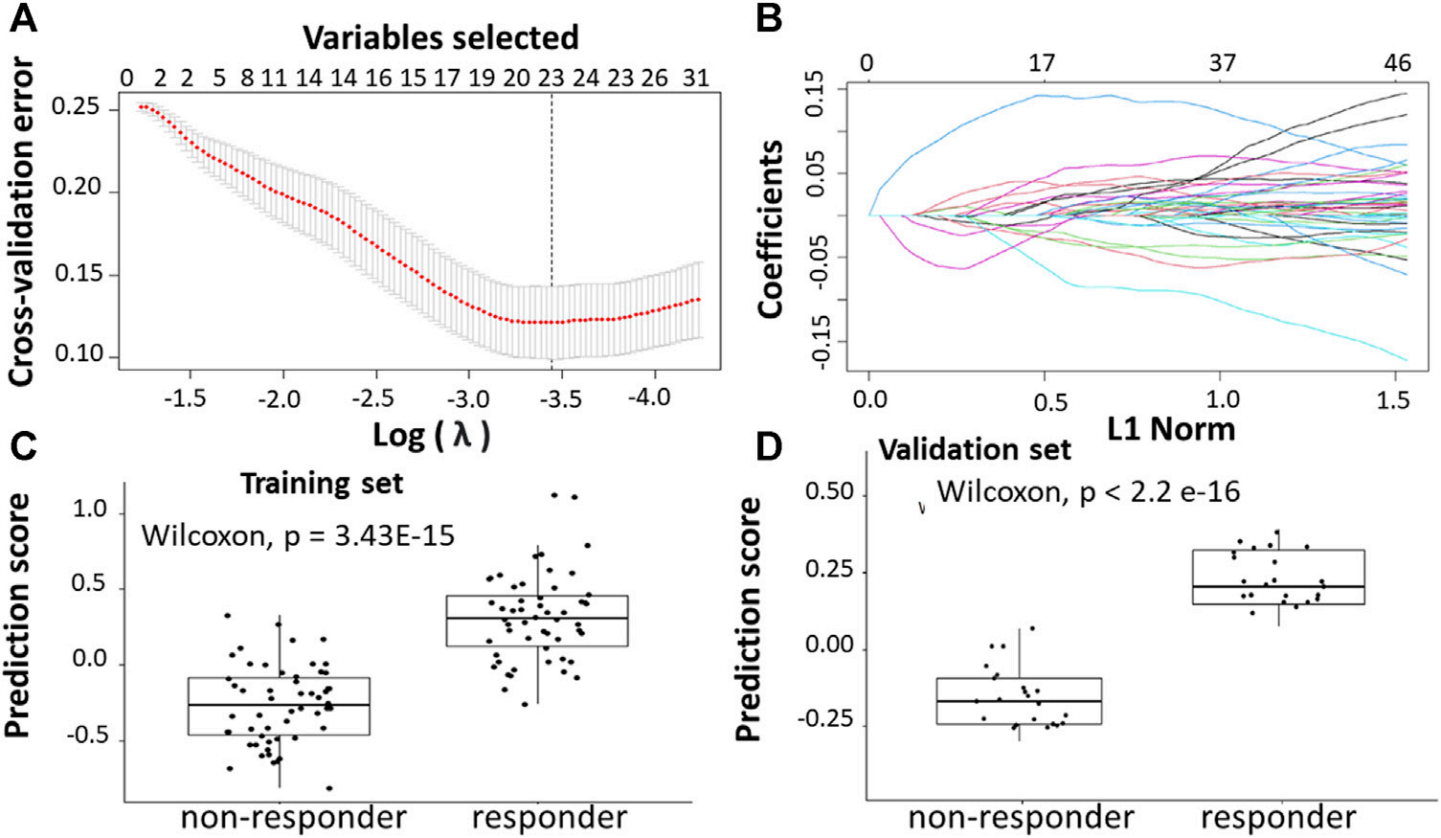
Construction of LASSO model. **(A)** Ten-fold cross-validation for tuning parameter selection in the LASSO model. **(B)** LASSO coefficient profiles of the training set. **(C)** The prediction score of the classifier (Equation 7) was higher in responder than in non-responder samples in the training set. **(D)** The prediction score of the classifier was higher in responder than non-responder samples in the validation set.

Following the identification of DEGs, the feature selection methods, LASSO and varSelRF, were applied to select gene signatures among DEGs. The LASSO method identified 23 genes that are relative to the drug response prediction. These genes were identified by selecting the optimal λ that was identified by performing the ten-fold cross-validations. The value of λ was determined by the minimum cross-validation error and was denoted as λmin. In this case, the λmin was equal to 0.0349, resulting in 23 non-zero coefficients including *TACSTD2, IFI44L, REEP1, WIF1, PPAT, IGF1, LY6G6D, CDKN1C, PPFIBP1, SFRP2, IFIT1, CMPK2, ZFTA, RETNLB, FER1L3,* HUNK*, GGTA1, ACSL6, LINC02067, LRRC69, RSAD2, LOC100507477, and MX1*. (Figures 4A,B). The expression of these genes was then used to evaluate the prediction score generated by the identified 23-genes that differentiate between the group of responders and non-responders. The following formula was used to calculate the prediction score of the identified genes:

$$\begin{array}{ll} Prediction\;Score & =\text{TACSTD}2\times 0.00270945684650097+\text{IQGAP}2\times \left(-0.00847808418322799\right)+\text{REEP}1\times 0.00605656057956512\\ & +\text{AKR}1B10\times \left(-0.0203366801255001\right)+\text{PPAT}\times 0.0201089896480733+\text{IGF}1\times \left(-0.0284545651292782\right)\\ & +\text{CDKN}1C\times 0.00741111664384363+\text{PPFIBP}1\times \left(-0.0317847003508003\right)+\text{EEF}1D\times 0.141850458165839\\ & +\text{BEX}4\times \left(-0.00176740773798336\right)+\text{PLEC}\times 0.0222352477700212+\text{ZFTA}\times 0.0102617253057372\\ & +\text{USH}1C\times 0.0545082812981942+\text{FER}1L4\times 0.0359442555529826+\text{ABI}3\text{BP}\times \left(-0.0755005720787851\right)\\ & +\text{GGTA}1\times \left(-0.00944239443492612\right)+\text{CREB}5\times 0.0351766533783486+\text{LINC}02067\times 0.00907100821483412\\ & +\text{LRRC}69\times 0.0238555594546346+\text{RAB}3\text{IP}\times 0.000169633620952202+\text{HSD}17B6\times \left(-0.00214863965806719\right) \end{array}$$ (7)

The results showed that these identified genes were able to differentiate between the group of responders and non-responders. As shown in the figure, the responders have higher prediction scores compared to the non-responders. This was also elucidated in the plot that represents the validation set (Figures 4C,D).

The varSelRF method identified 14 genes including *IFI44L, WIF1, IGF1, LY6G6D, CDKN1C, SFRP2, IFIT1, CMPK2, RETNLB,* HUNK*, ACSL6, RSAD2, LOC100507477, and MX1*. Using these methods, the genes were continuously evaluated. The gene set with best prediction performance used as the optimal gene set for further analysis. Twelve genes were identified as relevant genes from both methods, including Interferon Induced Protein 44 Like (***IFI44L***), WNT Inhibitory Factor 1 (***WIF1***), Lymphocyte Antigen 6 Family Member G6D (***LY6G6D***), Secreted Frizzled Related Protein 2 (***SFRP2***), Resistin Like Beta (***RETNLB***), Cytidine/Uridine Monophosphate Kinase 2 (***CMPK2***), Acyl-CoA Synthetase Long Chain Family Member 6 (***ACSL6***), Radical S-Adenosyl Methionine Domain Containing 2 (***RSAD2***), and lncRNA **(*LOC100507477*)**, Interferon Induced Protein With Tetratricopeptide Repeats 1 (***IFIT1***), MX Dynamin Like GTPase 1 **(*MX1*)**, Hormonally Upregulated Neu-Associated Kinase (HUNK).

The assessment of model performance was performed in training and validation sets according to accuracy, sensitivity, specificity, and AUC. As shown in Table 4, the top machine learning algorithm was random forest.

**TABLE 4:** Comparison of different classification methods on training and validation sets after features selection using LASSO and VarSelRF method.

Model		mFOLFOX (LASSO & VarSelRF)	
		Random forest (RF)	Support vector machine (SVM)
Training (n = 60)	Accuracy	1	0.96
	95% CI	(0.94, 1)	(0.84, 0.99)
	Sensitivity	1	1
	Specificity	1	0.86
Validation (n = 16)	Accuracy	0.93	0.91
	95% CI	(0.74, 0.94)	(0.8303, 0.95)
	Sensitivity	1	0.9
	Specificity	0.87	0.83
	AUC	0.92	0.91

For the training set, random forest algorithm achieved an accuracy of 1 with 95% CI ranging between 0.94 and 1. The sensitivity and specificity were equal to 1. Support vector machine, on the other hand, achieved an accuracy of 0.96 with 95% CI ranging between 0.84 and 0.99. The sensitivity and specificity are equal to 0.86 and 0.91 respectively (Table 4).

For the validation set, random forest algorithm had an accuracy of 0.93 with 95% CI ranging between 0.74 and 0.94. The sensitivity, specificity, and area under curve (AUC) are equal to 0.1, 0.87, and 0.92 respectively. The support vector machine algorithm achieved an accuracy of 0.91 with 95% CI ranging between 0.83 and 0.95. The sensitivity, specificity, and AUC are equal to 0.9, 0.83, 0.91 respectively (Table 4).

The protein-protein interaction (PPI) networks generated through IMEx indicate (direct and indirect) interactions among these gene encoded proteins (Supplementary Figure S3). As shown in Supplementary Figure S3, the PPI network comprise 92 nodes and 93 edges. 4 out of 12 genes formed hub genes. For instance, *IFIT1*, *MX1*, and *HUNK* had the highest number of protein interactions (Supplementary Figure S3). Based on the PPI network predicted using IMEx, the signature proteins have no known direct functional effect on each other. IFIT1 connects to *MX1* via *ISG15* and *IRF3*. On the other hand, *IFIT1* connects to *RSAD2* via *IRF9*, *CDK9*, *POLR2F* and *STAT1*. In addition, *HUNK*, *LEFTY1*, *CMPK2*, *RETNLB*, *SFRP2*, *WIF1*, and *MX1* interact directly with *UBC*.

### 3.4 Responders vs. non-responders samples who received FOLFIRI chemotherapy

The fourth analysis of colorectal cancer patients identified significant genes separating FOLFIRI responders from non-responders for metastatic stages of cancer. The training set, and validation set consisted of 66 and 15 CRC patients, respectively, from the combined dataset (GSE62080 and GSE72970) derived from GPL570 for patients who received first-line FOLFIRI-based treatment. These datasets included samples for a total of 45 non-responders and 36 responders of metastatic CRC samples. The independent test data included 57 patients (31 non-responders and 26 responders) from the dataset GSE62321 derived from the platform Affymetrix Human Genome U133B Array (GPL97).

**FIGURE 5:**
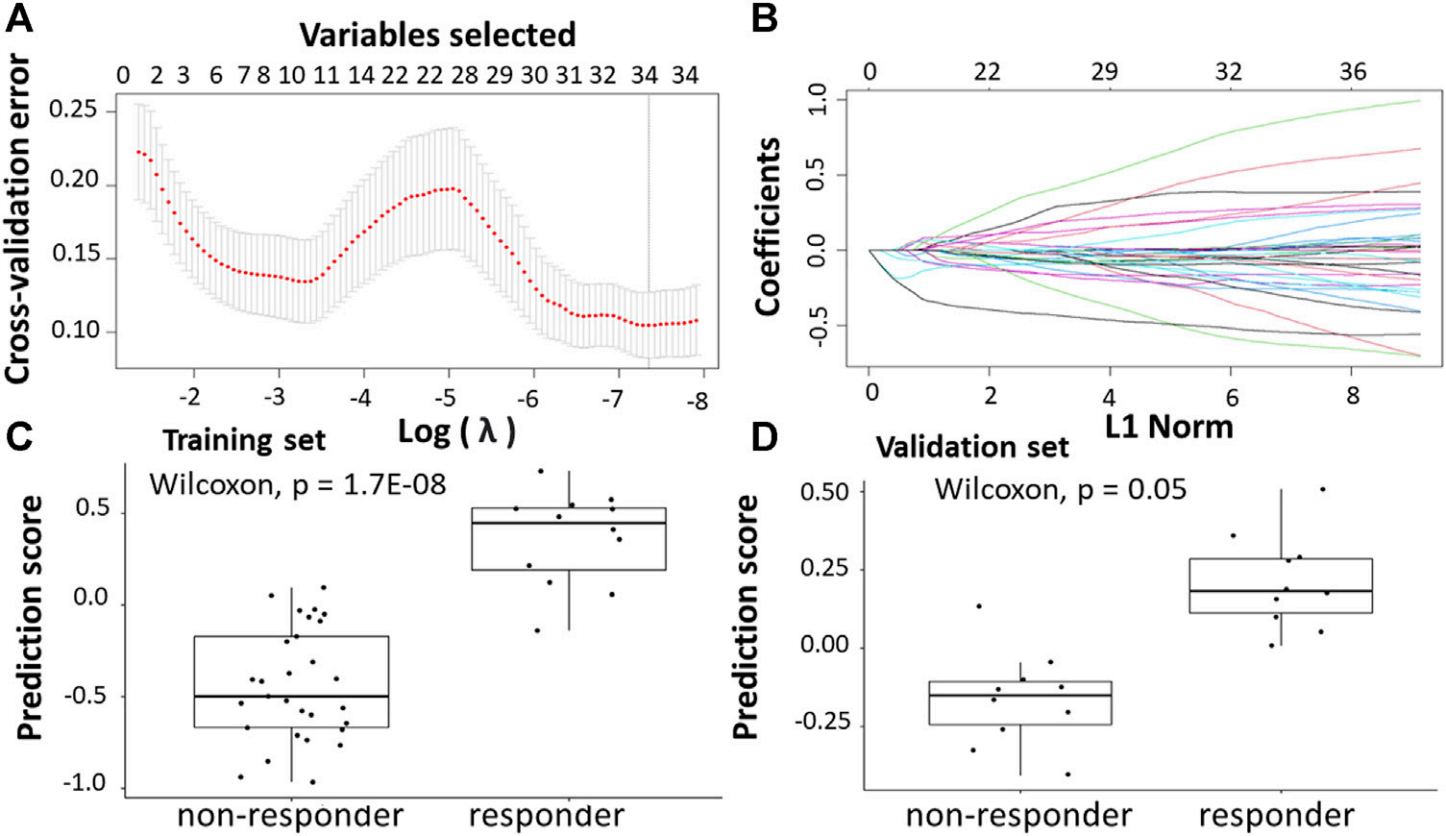
Construction of LASSO model. **(A)** Ten-fold cross-validation for tuning parameter selection in the LASSO model. **(B)** LASSO coefficient profiles of the training set. **(C)** The prediction score of the classifier (Equation 8) was higher in responder than in non-responder samples in the training set. **(D)** The prediction score of the classifier was higher in responder than non-responder samples in the validation set.

Following the identification of DEGs, the feature selection methods, LASSO and varSelRF, were applied to select gene signatures among DEGs. The LASSO method identified 34 genes that are relative to the drug response prediction. These genes were identified by selecting the optimal λ that was identified by performing the ten-fold cross-validations. The value of λ was determined by the minimum cross-validation error and was denoted as λmin. In this case, the λmin was equal to 0.0210, resulting in 34 non-zero coefficients including *OGN*, *NRP2*, *SFRP2*, *ABI3BP*, *MND1*, *CTHRC1*, *FBXO32*, *AMOTL1*, *RNA45SN5*, *DDR2*, *BOC*, *MAP1B*, *CLMP*, *FNDC1*, *GLT8D2*, *SLIT2*, *PAX8*. *AS1*, *AKAP12*, *CACNA2D1*, *PRKG1*, *PCDH7*, *CD36*, *COL1A2*, *LINC01614*, *LEMD1*, *PI15*, *PTGR2*, *COL3A1*, *RNF183*, *MIX23*, *CDH11*, *C3orf80*, and *SERPINB9* (Figures 5A,B). The expression of these genes was then used to evaluate the prediction score generated by the identified 34-genes that differentiate between the group of responders and non-responders. The following formula was used to calculate the prediction score of the identified genes:

$$\begin{array}{ll} Prediction\;Score & =\text{OGN}\times \left(-0.026747485\right)+\text{NRP}2\times \left(-0.479830846\right)+\text{SFRP}2\times \left(-0.008852721\right)+\text{ABI}3\text{BP}\times \left(-0.251862481\right)\\ & +\text{MND}1\times \left(-0.189794013\right)+\text{SLIT}2\times \left(-0.158823987\right)+\text{FBXO}32\times 0.378204031+\text{RNA}45\text{SN}5\times \left(-0.054747404\right)\\ & +\text{CAB}39L\times 0.249095948+\text{BOC}\times \left(-0.077415537\right)+\text{MAP}1B\times 0.301174128+\text{CLMP}\times 0.052730754\\ & +\text{FNDC}1\times \left(-0.023013935\right)+\text{GLT}8D2\times \left(-0.21784962\right)+\text{AMOTL}1\times 0.018417206+\text{PAX}8.\text{AS}1\times \left(-0.04543003\right)\\ & +\text{AKAP}12\times 0.876734016+\text{CACNA}2D1\times 0.12242411+COL1A2\times 0.018402322+\text{PCDH}7\times \left(-0.318842155\right)\\ & +\text{CD}36\times \left(-0.104574686\right)+\text{LINC}01614\times 0.039000746+\text{LEMD}1\times \left(-0.228052347\right)+\text{PI}15\times 0.005342257\\ & +\text{PTGR}2\times \left(-0.548660753\right)+\text{COL}3A1\times 0.588575905+\text{RNF}183\times 0.035670237+\text{DDR}2\times 0.02476893\\ & +\text{CDH}11\times 0.232601528+C3\text{orf}80\times 0.285287459+\text{CTHRC}1\times \left(-0.092173245\right) \end{array}$$ (8)

The results showed that these identified genes were able to differentiate between the group of responders and non-responders. As shown in the figure, the responders have higher prediction scores compared to the non-responders. This was also elucidated in the plot that represents the validation set (Figures 5C,D).

The varSelRF method identified 14 genes including *SFRP2*, *MND1*, *CTHRC1*, *AMOTL1*, *DDR2*, *FNDC1*, *GLT8D2*, *SLIT2*, *AKAP12*, *CD36*, *COL1A2*, *PTGR2*, *COL3A1*, *CDH11*. Using these methods, twelve genes were identified including Angiomotin Like 1 (***AMOTL1***), Collagen Triple Helix Repeat Containing 1 (***CTHRC1***), Fibronectin Type III Domain Containing 1 (***FNDC1***), Collagen Type I Alpha 2 Chain (***COL1A2***), Discoidin Domain Receptor Tyrosine Kinase 2 (***DDR2***), Slit homolog 2 (***SLIT2***), Cadherin 11 (***CDH11***), Collagen Type III Alpha 1 Chain (***COL3A1***), A-Kinase Anchoring Protein 12 (***AKAP12***), Secreted Frizzled Related Protein 2 (***SFRP2***), Prostaglandin Reductase 2 (***PTGR2***), and cluster of differentiation 36 (***CD36***).

The assessment of model performance was performed in training and validation sets according to accuracy, sensitivity, specificity, and AUC. As shown in Table 5, the top machine learning algorithm was random forest.

**TABLE 5:** Comparison of different classification methods on training, validation, and independent test set after feature selection using LASSO and VarSelRF method.

Model		mFOLFIRI (LASSO & VarSelRF)	
		Random forest (RF)	Support vector machine (SVM)
Training (n = 66)	Accuracy	1	0.96
	95% CI	(0.94, 1)	(0.84, 0.99)
	Sensitivity	1	1
	Specificity	1	0.86
Validation (n = 15)	Accuracy	0.93	0.93
	95% CI	(0.74, 0.94)	(0.83, 0.95)
	Sensitivity	1	0.95
	Specificity	0.87	0.86
	AUC	0.93	0.93
Independent Test (n = 57)	Accuracy	0.96	0.96
	95% CI	(0.87, 0.99)	(0.87, 0.99)
	Sensitivity	0.89	0.89
	Specificity	0.92	0.93
	AUC	0.94	0.93

For the training set, random forest algorithm achieved an accuracy of 1 with 95% CI ranging between 0.94 and 1. The sensitivity and specificity were equal to 1. Support vector machine, on the other hand, achieved an accuracy of 0.96 with 95% CI ranging between 0.84 and 0.99. The sensitivity and specificity are equal to 0.86 and 0.93 respectively (Table 5).

For the validation set, random forest algorithm had an accuracy of 0.93 with 95% CI ranging between 0.74 and 0.94. The sensitivity, specificity, and area under curve (AUC) are equal to 1, 0.87, and 0.93 respectively. The support vector machine algorithm achieved an accuracy of 0.93 with 95% CI ranging between 0.83 and 0.95. The sensitivity, specificity, and AUC are equal to 0.95, 0.86, 0.93 respectively (Table 5).

For the validation set, random forest algorithm had an accuracy of 0.96 with 95% CI ranging between 0.87 and 0.99. The sensitivity, specificity, and area under curve (AUC) are equal to 0.89, 0.92, and 0.94 respectively. The support vector machine algorithm achieved an accuracy of 0.96 with 95% CI ranging between 0.87 and 0.99. The sensitivity, specificity, and AUC are equal to 0.89, 0.93, 0.93 respectively (Table 5).

The protein-protein interaction (PPI) networks generated through IMEx indicate (direct and indirect) interactions among these gene encoded proteins (Supplementary Figure S4). As shown in Supplementary Figure S4, the PPI network comprises 114 nodes and 112 edges with 5 out of 10 genes being hub genes. For instance, *CD36*, *AKAP12*, *AMOTL1*, and *COL1A2* have the highest number of hub genes. Based on the PPI network predicted using IMEx, the signature proteins have no known direct functional effect on each other. CD36 connects to *COL1A2* via *PXN* gene encoded protein. *COL3A1* also connects to *COL1A2* via *SP1*, *MYOC*, *FMOD*, *ASPN*, and *MXRA5*. *AKAP12* connects to *CDH11* via *CTNNB1* and to *DDR2* via EGFR. In addition, *SFRP2*, *SLIT2*, *AKAP12*, and *CD11* interact directly with CTNNB1. On the other hand, *AMOTL1*, *COL1A2*, *DDR2*, *AKAP12*, and *SLIT2* interact directly with *UBC*.

### 3.5 Machine learning model application to predict effectiveness of alternate chemotherapy regimen

In the analysis above, the genes that successfully classified responders and non-responders for FOLFOX differed from the genes that successfully classified responders and non-responders for FOLFIRI except for one gene that was present in both, namely, secreted frizzled related protein 2 (SFRP2). This suggests that there might be different underlying mechanisms involved (consistent with the two therapies differing in cellular targets) and, consequently, patients who did not respond to FOLFOX might respond to FOLFIRI and *vice versa*. When the Random Forest model for the FOLFIRI data set was applied to the prediction of cases of colon cancer treated with the FOLFOX regimen, the results show that 25 of 56 (44.6%) primary CRC patients who did not respond to FOLFOX would respond to FOLFIRI and that 20 of 76 samples (26.3%) of metastatic CRC patients who did not respond to FOLFOX are predicted to respond to FOLFIRI (Table 6). When the FOLFOX training model for metastatic CRC was applied to the prediction of cases of colon cancer samples treated with the FOLFIRI regimen, the results showed 25 of 81 (30.9%) patients who did not respond to FOLFIRI would respond to FOLFOX. Applying the FOLFOX training model for primary CRC to the FOLFIRI cases, 5 of 81 (6.2%) patients who did not respond to FOLFIRI are predicted to respond to FOLFOX. Assuming 94% accuracy for the FOLFOX model and 96% accuracy for the FOLFIRI model, a Chi-squared test shows that these results are significant at the *p* > 0.00001 level. This analysis predicts that it is likely that 28.6% of patients on average that failed one drug treatment regimen would have responded to the other treatment regimen. However, further clinical validation would be needed before this could influence clinical care.

**TABLE 6:** Prediction of alternative therapy efficacy.

Machine Learning Model	Patient Data	
	FOLFOX responder(Primary)	FOLFOX non-responder(Primary)
**Responder with FOLFIRI model**	8 (14.3%)	**25 (44.6%)**
**Non-responder with FOLFIRI model**	23 (41.8%)	0 (0.0%)
	FOLFOX responder(metastasis)	FOLFOX non-responder(metastasis)
**Responder with FOLFIRI model**	15 (19.7%)	**20 (26.3%)**
**Non-responder with FOLFIRI model**	19 (25%)	22 (28.9%)
	FOLFIRI responder	FOLFIRI non-responder
**Responder with FOLFOX** **(metastasis) model**	12 (14.8%)	**25 (30.09%)**
**Non-responder with FOLFOX** **(metastasis) model**	24 (29.6%)	20 (24.7%)
	FOLFIRI responder	FOLFIRI non-responder
**Responder with FOLFOX** **(primary) model**	19 (23.4%)	**5 (6.2%)**
**Non-responder with FOLFOX** **(primary) model**	26 (32.09%)	40 (49.3%)

The numbers in bold indicate the patients that would have responded to the alternate therapy as predicted by the model.

## 4 Discussion

FOLFOX and FOLFIRI are combination chemotherapies that have been used as a first-line treatment for patients with late-stage colon cancer. Previous studies have shown FOLFOX and FOLFIRI to be ∼52% and ∼39% effective, respectively (Giacchetti et al., 2000; Neugut et al., 2019). Though these regimens can significantly extend the median overall survival up to 15 months, many individuals do not achieve long-term clinical benefit with a given treatment (Goldberg, 2006). Since these therapies target different cell mechanisms, there is the possibility that the actual responders may be different between the two drugs. Thus, improving methods of identifying patients who would respond better to these drugs would help oncologists determine optimum treatment regimens for their patients. It is important to determine whether or not the patient will respond to the chemotherapy treatment not only to increase survival but also to minimize the sometimes severe side effects of agents such as FOLFOX and FOLFIRI.

Gene-expression profiles have the potential to predict cancer patient outcome and drug response in comparison to the conventional clinical and pathological techniques (Gordon et al., 2003; Nutt et al., 2003; Hess et al., 2006; Del Rio et al., 2007; Parissenti et al., 2007). In contrast to the numerous studies to identify the estimation of responders to anticancer drugs using expression profiling in other cancer types such breast and ovarian cancer, only a few such studies have been conducted in colorectal cancer (Del Rio et al., 2007; Nannini et al., 2009; Tsuji et al., 2012; Lu et al., 2020). A direct comparison with a previously published machine learning model on the same dataset indicates that the performance of the models presented in this paper is superior in predicting FOLFOX and/or FOLFIRI drug response. Tsuji and co-workers identified a signature consisting of 14 genes using random forest embedded selection that was able to predict FOLFOX responders in a sample size of 83 patients (Tsuji et al., 2012). Using these genes, RF classifier was able to correctly classify 21 of 23 responders (91.3%) and 22 of 23 non-responders (95.6%) in the training set, with an accuracy of 69.2% in 29 independent test samples (Tsuji et al., 2012). Also, an older study by Del Rio and co-workers identified 14 genes for predicting response to FOLFIRI, although it included only 21 patients (Del Rio et al., 2007).

The purpose of this study was to identify gene signatures that could predict the response to FOLFOX and FOLFIRI in patients with early stage and metastatic CRC. To determine the gene signature for response prediction from gene expression profiling, significant differentially expressed genes (DEGs) were first selected. The DEGs were filtered using the variable selection methods including LASSO and varSelRF. The performance of the models was evaluated using two machine learning classifiers, RF and SVM. Overall, the machine learning model with enhanced feature selection achieved 94%–96% accuracy for predicting the response of patients to FOLFOX or FOLFIRI using retrospective cancer patient data available in public datasets. These results were held for data sets that were not part of the training data. Furthermore, for those patients that did not respond to FOLFOX, 35% are predicted as FOLFIRI responders and for those patients that did not respond to FOLFIRI 18% are predicted as FOLFOX responders. This suggests that the biomarkers identified here can help select which chemotherapy regimen to use on patients after additional validation studies.

### 4.1 Random forests machine learning models outperform SVM in these studies

In the machine learning analysis, the random forest models performed better than the support vector machine models in almost all models. The machine learning literature specifies that random forests handle noisy data and outliers better than SVMs (Cherkassky and Ma, 2004; Goldstein et al., 2011; Wang and Li, 2017; Sabzekar and Hasheminejad, 2021). This is due to several properties of the random forest method as described by Brieman in 2001 (Breiman, 2001). First, random forest has been identified as the best method for low sample size and a large number of features (Breiman, 2001). Second, Brieman demonstrated that random forests do not overfit with and increasing number of trees. The accuracy simply stops increasing as the number of trees increases Finally, random forests were described to be robust with respect to noise and that randomness in large data sets can actually increases accuracy in classification in contrast to regression where randomness can decrease accuracy. In addition, while both algorithms can model non-linear relationships, random forest excels in naturally capturing these relationships compared to SVM (Cherkassky and Ma, 2004; Goldstein et al., 2011; Wang and Li, 2017; Sabzekar and Hasheminejad, 2021). The latter achieves non-linearity using kernel functions, a process that can sometimes pose challenges in selecting the appropriate kernel and tuning its parameters (Cherkassky and Ma, 2004; Sabzekar and Hasheminejad, 2021). Furthermore, random forests tend to demonstrate robust performance with small size datasets, while SVMs might require a larger volume of data to achieve effective generalization, especially when dealing with complex, high-dimensional problems (Cherkassky and Ma, 2004; Goldstein et al., 2011; Wang and Li, 2017; Sabzekar and Hasheminejad, 2021).

### 4.2 Differences in gene signatures

The chemotherapy agent FOLFOX consists of leucovorin calcium (folinic acid), fluorouracil, and oxaliplatin. FOLFIRI on the other hand consists of leucovorin calcium (folinic acid), fluorouracil, and irinotecan. Leucovorin enhances fluorouracil binding and inhibition of thymidylate synthase (Rustum, 1990). Thymidylate synthase is critical for the synthesis of 2′-deoxythymidine-5′-monophosphate which is need for DNA synthesis (Rose et al., 2002). Oxaliplatin binds DNA to disrupt DNA synthesis and transcription (Arango et al., 2004). Histone H3 is coupled to DNA synthesis (Tagami et al., 2004). In the FOLFOX gene expression Histone H3 is activated and serves as a hub for interactions for many of the proteins that are under expressed in responders vs. non-responders. The patients that are predicted to be sensitive to FOLFOX have enhanced DNA synthesis through active histone H3 and the oxaliplatin mitigates this effect through disruption of DNA synthesis. Irinotecan targets topoisomerase I which is essential for proper DNA topology during replication and transcription (Kciuk et al., 2020). Topoisomerase I interacts with c-Jun which is involved cell proliferation observed in colorectal cancer (Kciuk et al., 2020). Analysis of the gene signatures used for classification in the machine learning models suggest how these individual genes relate to chemotherapy response.

**FIGURE 6:**
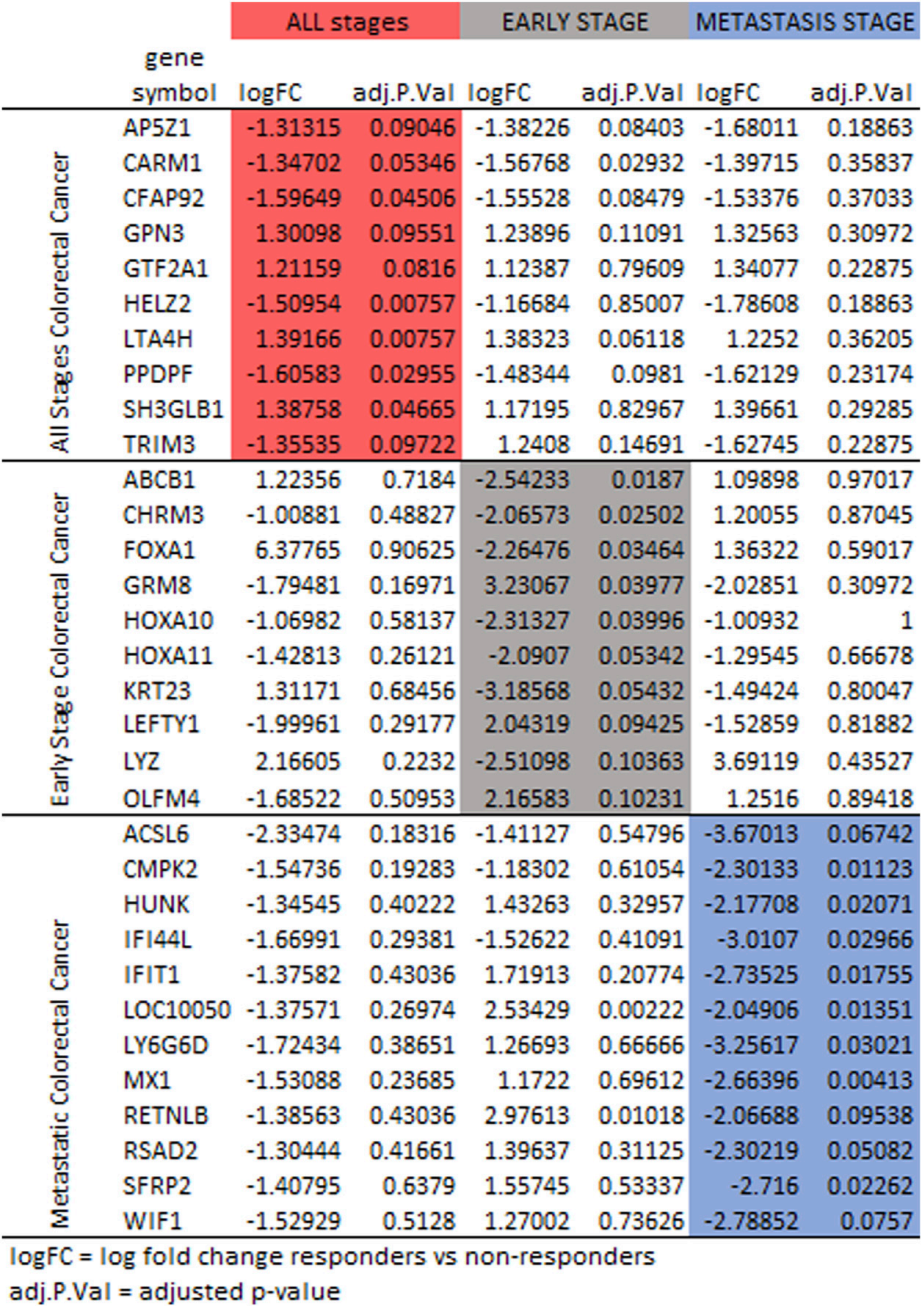
Protein expression for the gene signatures for FOLFOX treatment for all stages (red), early-stage (gray), and metastatic (blue) colorectal cancer. Shown are log fold change and adjusted *p*-value (*p* < 0.10 is significant).

Differences were observed in the genes selected to classify responder to non-responder for FOLFOX when comparing patients with all stages of colorectal cancer, patients with early-stage colorectal cancer and patients with metastatic colorectal cancer. It has been established that the gene expression profiles differ for early-stage and metastatic colorectal cancer (Poturnajova et al., 2021; Peixoto et al., 2023). Figure 6 compares the log fold change and adjusted *p*-values for genes selected. For some genes the three cohorts (all stages, early-stage, metastatic) follow the same trend of upregulation or downregulation. In other cases, early-stage and metastatic log fold change are quite different. The machine learning feature selection simply chooses the genes best suited to classify responder vs. non-responder in each data set. While the machine learning does not use the adjusted *p*-value specifically, Figure 6 shows that in most cases the value is above the 0.10 threshold for significance in the cohorts other than the one that used the gene in gene signature. There are some genes that have very log fold change values between patients with early-stage colorectal cancer and patients with metastatic colorectal cancer such as TRIM3, ABCB1, FOXA1, GRM8, LEFTY1, LYZ, HUNK, IFIT1, LY6G6D, MX1, RETNLB, RSAD2, SRFP2, and WIF1.

### 4.3 Biological significance of FOLFOX gene signatures

**FIGURE 7:**
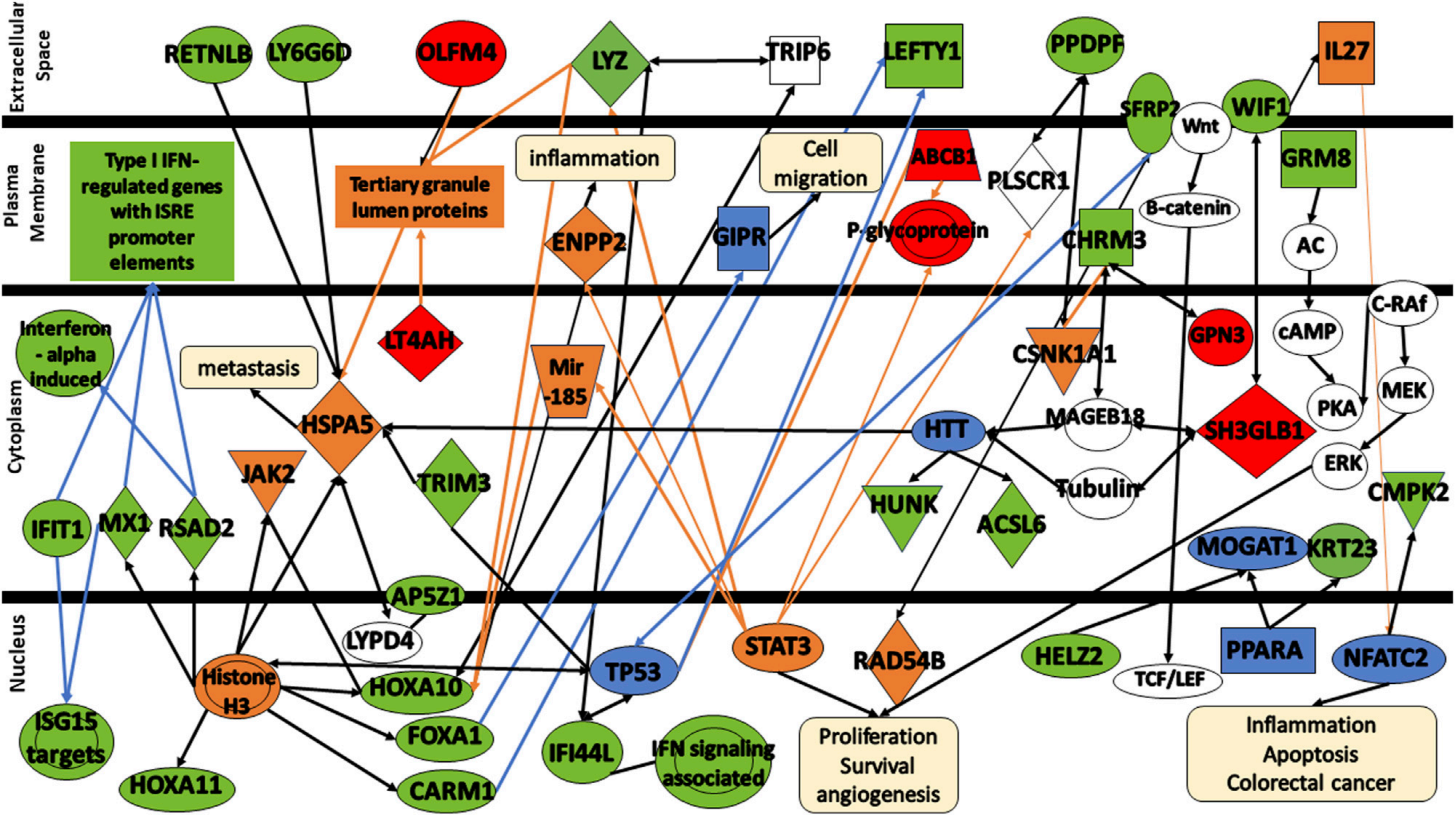
Protein signaling pathways of the identified gene signatures in the colorectal cancer on the response of colorectal cancer patients to FOLFOX drugs. Green color represents gene signatures under expression; red color represents gene signatures over expression; orange color represents prediction of molecule activation; dashed lines represent indirect relationship; solid lines represent direct relationship. Abbreviations: **RETNLB**, Resistin Like Beta; **LY6G6D**, Lymphocyte antigen 6 complex locus G6D; **OLFM4**, Olfactomedin-4; **IFIT1**, Interferon-induced Protein with Tetratricopeptide Repeats 1; **MX1**, MX Dynamin Like GTPase 1; **RSAD2**, Radical S-adenosyl methionine domain containing 2. **HOXA11**, Homeobox A11; **HOXA10**, Homeobox A10; **FOXA1**, Forkhead box protein A1; **CARM1**, Coactivator-associated arginine methyltransferase 1; **AP5Z1**, AP-5 complex subunit zeta; **IFI44**, Interferon induced protein 44; **LYZ**, lysozyme; **LT4AH**, Leukotriene A4 Hydrolase; **SFRP2**, secreted frizzled related protein 2; **LEFTY1**, left-right determination factor 1; **PPDPF**, pancreatic progenitor cell differentiation and proliferation factor; **WIF1**, WNT Inhibitory Factor 1; **CHRM3**, cholinergic receptor muscarinic 3; **GRM8**, glutamate metabotropic receptor 8; **HUNK**, hormonally upregulated Neu-associated kinase; **ACSL6**, acyl-coenzyme A synthetase long-chain family member 6; **CMPK2**, cytidine/uridine monophosphate kinase 2; **HELZ2**, helicase with zinc finger 2; **GPN3**, GPN-Loop GTPase 3; **SH3GLB1**, SH3 Domain Containing GRB2 Like, Endophilin B1; **KRT23**, keratin 23; **TRIM3**, Tripartite Motif Containing 3.

To obtain a better understanding of the biological significance of the 32 DEGs in colorectal cancer, the gene signatures were subjected to IPA software library was used to generate a schematic network of gene signatures in different signaling pathways, elucidating their effect on the response of colorectal cancer patients to FOLFOX drug (Figure 7). HOXA10, HOXA11, FOXA1, CARM1, RSAD2, and MX1 are activated via the promotion of Histone H3. Both Histone H3 and HOXA10 are linked to the activation of JAK2. HOXA10 can also be activated via the signal LYZ and mir-185. FOXA1 is associated with TRIP6 which seems to be linked to LYZ. In addition, FOXA1 appears to be involved in the inhibition of GIPR, a factor crucial for cell migration inhibition. Both OLFM4, LT4AH, and LYZ are linked to tertiary granule lumen proteins. OLFM4, Histone H3, HTT activate HSPA5, an important molecule that tends to lead to cancer metastasis. HSPA5 is associated with RETNLB, LY6G6D, AP5Z1 via LYPD4, and TRIM3. Both TRIM3 and Histone H3 are associated with TP53. In addition, both CARM1 and TP53 seem to be involved in the decrease of LEFTY1 expression. TP53 along with RAD54B are linked to the decrease of SFRP2 expression. HTT is associated with various gene signatures including HUNK, ACSL6, SH3GLB1 via both MAGEB18 or Tubulin, and CHRM3 via MAGEB18. CHRM3 is also linked to the expression of GPN3 and CSNK1A1. CSNK1A1, in turn, is associated with PPDPF. SH3BL1 is linked to WIF1, activating IL27 and influencing NFATC2 and CMPK2. NFATC2 is involved in many mechanisms including inflammation, apoptosis, and colorectal cancer. STAT3 is involved in the expression of many molecules including ABCB1, LYZ, ENPP2, mir-185, P-glycoprotein, and PLSCR1. STAT3 is involved in many cellular functions including cell proliferation, survival, and angiogenesis. Finally, Both HELZ2 and KRT23 are expressed via PRAPA.

Further analysis of the changes in the expression levels of gene signatures shed some insights into the mechanisms involved and differences in response to FOLFOX between early stage and metastatic colorectal cancer.

CARM1—also known as *PRMT4*, acts as a transcriptional coactivator for several different types of DNA-binding transcriptional activator proteins, and thus deregulated *CARM1* expression likely to affect many transcriptional programs which target genes that control proliferation rate or other oncogenic properties (Hong et al., 2004; Frietze et al., 2008; Chen et al., 2009; Kim et al., 2010; Ou et al., 2011). Activation of the Wnt/β-catenin and inflammatory signaling pathways disrupts intestinal epithelial homeostasis, resulting in increased proliferation, decreased differentiation, and decreased apoptosis (Grivennikov, 2013). The reduced expression of CARM1 seen in responders at all stages of colorectal cancer compared with non-responders might reflex reduction in Wnt signaling reducing chemoresistance making the cells more susceptible to FOLFOX.

GPN3—GPN-Loop GTPase 3 (GPN3) has been shown to be essential for proliferation in breast cancer (Lara-Chacón et al., 2019). It is upregulated in the all stages responders to FOLFOX compared to non-resonders. These cancer cells might display stronger proliferation and therefore be more susceptible to FOFOZX chemotherapy.

GTF2A1—General transcription factor IIA subunit 1 (GTF2A1) plays a role in DNA transcription and is part of RNA polymerase II initiation complex. It is upregulated in the all stages FOLFOX responders vs. non-responders. This might indicate that these colorectal cancer cells might be more transcriptionally active suggesting the cells are proliferating making them more susceptible to FOLFOX.

LTA4H–*LTA4H* (leukotriene A4 hydrolase) is an epoxide hydrolase that catalyzes conversion of the unstable allelic epoxide LTA4 to leukotriene B4 (LTB4) (Zhao et al., 2019). *LTA4H is* overexpressed in several cancers including CRC, and several studies have shown that its hydrolase function is implicated in cancer development (Ihara et al., 2007; Jeong et al., 2009; Teixeira and Sousa, 2022). *LTA4H* is a key modulator of the cell cycle through its negative effect on the expression of the tumor suppressor p27 protein (Oi et al., 2017; Teixeira and Sousa, 2022). Inhibtion of Leucotriene A4 hydrolase (LTA4H) reduces cellular proliferation in colorectal cancer (Zhao et al., 2019). The responders LTA4H expression is upregulated compared to non-responders suggesting increased cellular proliferation making them more susceptible to FOLFOX.

PPDPF–Pancreatic Progenitor Cell Differentiation And Proliferation Factor (PPDPF) overexpression has been observed to suppress mTOR signaling (Ma et al., 2021). In the all stage responders to FOLFOX compared to the non-responders PPDPF is downregulated relieving suppression of the mTOR pathway making the way for growth and proliferation.

SH3GLB1—*SH3GLB1* (SH3 domain GRB2-like endophilin B1), also known as Bif-1 and endophilin B1, is a tumor suppressor gene of the endophilin protein family (Pierrat et al., 2001; Snoek et al., 2008; Mokarram et al., 2017). SH3GLB1 interacts with BAX to regulate apoptosis (Cuddeback et al., 2001). Inhibition of SH3GLB1 suppresses apoptotic cell death by inhibiting BAX-BAK1 conformational change and caspase activation (Takahashi et al., 2005; Mokarram et al., 2017). Reduced expression of Bax was correlated with poor differentiation, metastatic progression, and is a negative prognostic factor in patients with CRC (Sturm et al., 1999; Jansson and Sun, 2002; Ko et al., 2013)It is upregulated in all stage FOLFOX responders compared non-responders. If apoptosis is favorable in these cells they will be more susceptible to FOLFOX.

TRIM3—TRIM3 is a tumor suppressor gene in colorectal cancer progression by stabilizing p53 another tumor suppressor and growth repressor (Piao et al., 2016). The TRIM3 expression log fold change (responders vs. non-responders to FOLFOX) −1.36 in all stages of cancer samples suggesting that the smaller amount of TRIM3 leads to more growth and proliferation which can make the cancer more susceptible to FOLFOX (Zhao, 2016). A recent study has been shown that TRIM3 inactivates the p38 MAPK pathway, which has negative effects on cell proliferation (Song et al., 2018). However, the results of the inactivation of p38 signaling pathway depend significantly on the cellular environment, and more specifically on the presence of a mutated or wildtype p53 (Gonzalez et al., 2022). In the former, *TRIM3* action contributes to chemoresistance to DNA-damaging drugs by suppressing apoptosis, whereas in the latter, it can suppress cell proliferation increasing the response to the chemotherapeutic agent (Sanchez-Prieto et al., 2000; Stramucci et al., 2018; Gonzalez et al., 2022).

ABCB1—ABCB1 is a transporter gene that has been implicated in cancer drug resistance. In the early stage samples where it serves as a biomarker the log fold change is 1.03 indicating that the cancer cells with less transporter are more susceptible to FOLFOX. ABCB1 gene was found to be highly expressed in CRC (Gottesman and Pastan, 2015). The expression of ABCB1 causes chemotherapy failure owing to the efflux of drug molecules out of the cancer cell (Linn and Giaccone, 1995; Beklen et al., 2020). It decreases the intracellular concentration of wide spectrum of hydrophobic, neutral, or positively charged drugs such as oxaplatin, taxanes and anthracyclines (O'Brien et al., 2007; Ricci-Vitiani et al., 2007; Nguyen et al., 2012). Further research is required to validate the link between ABCB1 and drug resistance in CRC.

FOXA1—FOXA1 inhibits anoikis (cell death upon detachment from the extracellular matrix) in colorectal cancer (Lazar et al., 2020). In the early stage cancer the log fold change of the responders vs. non-responders is −2.09. Resistance to anoikis has been associated with resistance to FOLFOX (Escalante et al., 2021). This lower amount of FOXAI in responders likely makes these cancers more susceptible to FOLFOX.

GRM8—GRM8 is a metabotropic glutamate receptor that is involved with the inhibition of cyclic AMP cascade and activating MAPK (Zhang et al., 2019). Activation of cAMP-PKA signaling mechanism promotes cancer growth, migration, metabolism and drug resistance, and invasion (Zhang et al., 2020a). Hence, the gene expression reduction in FOLFOX responders vs. non-responders (logFC = −1.17) might reduce drug resistance in early stage colorectal cancer.

KRT23—Keratin23 (KRT23) is a cytoskeletal protein. KRT23 knockdown decreased DNA damage repair in colorectal cancer cells (Birkenkamp-Demtröder et al., 2013). In the FOLFOX early-stage responders the KRT23 expression is downregulated compared to non-responders. The deficiency in DNA damage repair might make the cells easier to kill with FOLFOX.

LEFTY1—LEFTY1 has been observed to promote growth as it codes for a ligand of TGF-β. The positive log fold change of 2.04 suggests that the early-stage cancers susceptible to FOLFOX have increased cell growth.

LYZ–LYZ encodes for lysozme that has been demonstration to be an anticancer agent by blocking proliferation (Khan et al., 2019). The reduction in lysozyme in early stage responders would lead to increased proliferation which as stated previously would likely increase susceptibility to chemotherapy. Further studies is needed to confirm the mechanism of LYZ in drug resistance.

OLFM4—Olfactomedin 4 (OLFM4) is a glycoprotein that is a marker for intestinal stem cells. Increase expression has been correlated with cancer progression, metastases, and gastrointestinal inflammation (Liu and Rodgers, 2016). OLFM4 is upregulated in FOLFOX early-stage responders compared to non-responders. It is not clear how OLFM4 relates to FOLFOX treatment.

ACSL6—ASL6 encodes a long-chain acyl-coenzyme A synthase that is involved in fatty acid anabolism (Quan et al., 2021). In cancers the fatty acid are involved in mediating between anabolic and catabolic pathways (Rossi Sebastiano and Konstantinidou, 2019). The decreased expression of ASCL6 in FOLFOX responders with metastatic colorectal cancer suggests that cancer will have less anabolic (and maybe more catabolic) metabolism than the non-responders which might indicate more energy metabolism suggesting rapid proliferation and more sensitivity to FOLFOX.

CMPK2—CMPK2 is a long non-coding RNA that is typically upregulated in colorectal cancer and is positively correlated with metastases to lymph nodes and advanced stages through stimulation of FUBP3–c-Myc signaling (Gao et al., 2020). Furthermore, it increases cell proliferation. FUBP3 promotes immune infiltration and inflammation (Li et al., 2022). The reduced expression of CMPK2 seen in the responders would result in less inflammation which results in better response to chemotherapy.

HUNK–HUNK suppresses cell proliferation in the intestine (Reed et al., 2015). In the metastatic cancer samples the log fold change is −2.18 indicating that there is removal of cell proliferation suppression in the responders. Once again the fast growing cells are more susceptible to FOLFOX. Furthermore, a previous study demonstrated that HUNK expression becomes significantly upregulated from the earliest stages of tumor initiation following Apc loss, indicating this gene is probably a Wnt signaling target gene (Reed et al., 2015).

IFIT1—The IFIT family protein has been observed to inhibit proliferation (Pidugu et al., 2019). The negative log fold change score of −2.73 indicates that IFIT1 is downregulated in responders suggesting that the suppression of proliferation is removed. IFITs play a crucial role in host antiviral defense as an innate immune response (Ohsugi et al., 2017). Expression of IFITs is induced by viral and bacterial infection, type I IFN including IFN-α/β, and a variety of cellular stresses such as DNA damage (Levy et al., 1986; Weaver et al., 1998; Andersen et al., 2008; Ohsugi et al., 2017).

IFI44L - Interferon Induced Protein 44 Like (IFI44L) is a tumor suppressor. Knock-down of IFI44L results in increased cell proliferation (Zeng et al., 2023). In the metastatic responders to FOLFOX, the expression is lower than the non-responders suggesting increase cell proliferation which would make chemotherapy more effective.

LY6G6D–LY6G6D expression has been linked with immune evasiveness of a cancer (Corrales et al., 2022). The responders to FOLFOX have reduced expression (logFC = −3.26) suggesting that they are more susceptible to the immune response. A recent study evaluated LY6G6D and CD15 as predictive biomarkers for the response to JAK- and MAPK-directed therapies and found that these two biomarkers promote chemo-immune-resistance in immunologically compromised colon cancers and can be used as biomarkers to decide patients treatments (Giordano et al., 2019).

MX1—The MX1 gene encodes a GTPase called MxA that inhibits motility and invasiveness of cancer. In the responders, colorectal cancers with high MX-1 tend to be more invasive with more metastases (Croner et al., 2014). The responders in the metastatic samples have reduced expression (logFC = −2.66) suggesting that these cancers are less aggressive and might have better outcomes to chemotherapy. The results are consistent with previous studies. Shimizu et al. (2010) identified *MX1* as one of the pro-apoptotic genes. The altered expression of genes that encode apoptotic proteins contribute to cell accumulation in the colon, promoting malignancy and subsequent metastasis, allow tumor cells to survive in a suspended state, and provide cells with inherent resistance to anticancer drugs (Shimizu et al., 2010).

RETNLB–RETNLB has been found to be overexpressed in ∼80% of colorectal cancer patients positively correlating with patient survival (Di Rosa et al., 2023). RETNLB has been found to associate with HSPA5 whose activation leads to metastasis. Several studies demonstrated that HSPA5, beyond its chaperoning function, it is a multifunctional protein that exerts critical roles in cell proliferation, apoptosis, and resistance to chemotherapy agents (Luo et al., 2016). The metastatic cancers that responded to FOLFOX had reduced expression of RETNLB compared to non-responders (logFC = −2.07). Reduced RETNLB has been linked to increased sugar uptake (Abaandou et al., 2021). This suggests that the cells have rapid metabolism making them more susceptible to chemotherapy.

RSAD2—RSAD2 is involved the cellular signal for the immune response and inflammation (Sun et al., 2022). A reduction in RSAD2 expression in metastatic responders compared to non-responders to FOLFOX (logFC = −2.30) is observed. Studies have shown that the use of anti-inflammatories in colorectal cancer reduces mortality (Sada et al., 2020). It is possible that the reduction in RSAD2 also leads to better outcomes.

SRFP2 - SRFP2 works with the Wnt/β-catenin signaling pathway to promote cell homeostasis and contribute to chemoresistance (Sun et al., 2016). Wnt/β-catenin signaling promotes drug resistance through sensitization of the ABCB1 transporter (Zhu et al., 2021). It has been reported that overexpression of SFRP2 promotes the expression of YAP1 and the overexpression of YAP1 and SFRP2 promote the expression of β-catenin in CRC cells (Bai et al., 2021). The metastatic responders showed reduced expression compared to non-responders to FOLFOX (logFC = −2.72). The lower SFRP2 likely abrogates chemoresistance signaling.

WIF1—WIF1 suppression the Wnt/β-catenin signaling pathway will reduce chemoresistance (Zhu et al., 2021). Therefore, The reduced expression in the responders (logFC = −2.79) suggests reduced chemoresistance.

LOC10050 - Long Intergenic Non-Protein Coding RNA 10050 (LOC10050) is a DNA repair gene. The reduced expression seen in FOLFOX metastatic responders compared to non-responders suggest that these cancers will have less effective DNA repair making them less viable under chemotherapy.

### 4.4 Biological significance of FOLFIRI gene signatures

**FIGURE 8:**
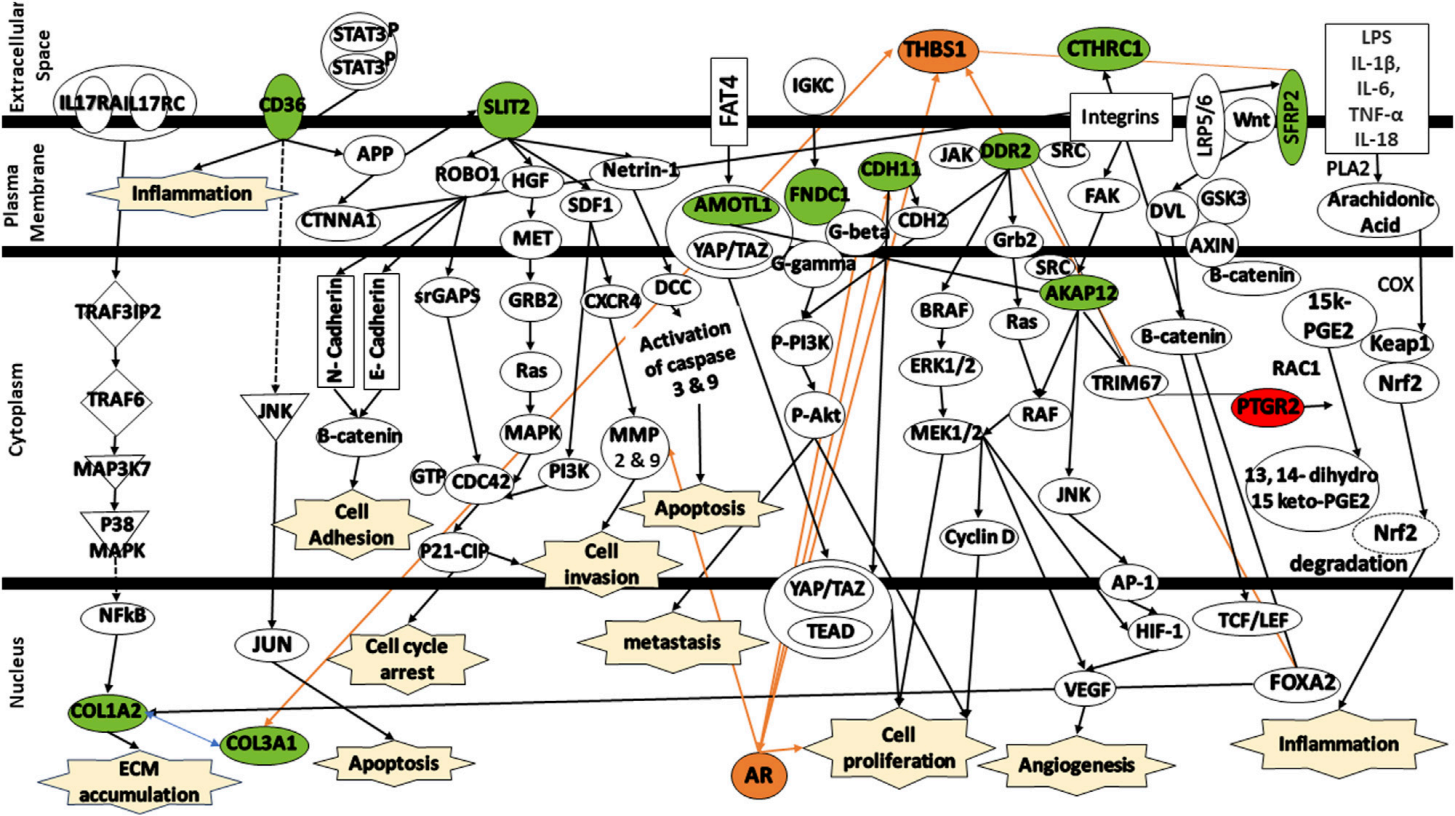
Protein signaling pathways of the identified gene signatures in the colorectal cancer on the response of colorectal cancer patients to FOLFIRI drugs. Green color represents under expression; red color represents over expression; orange color represents prediction of molecule activation; dashed lines represent indirect relationship; solid lines represent direct relationship. Abbreviations: **CD36**, Cluster of differentiation 36; **COL1A2**, Collagen type I alpha 2 chain; **COL3A1**, Collagen type III alpha 1 chain; **SLIT2**, Slit guidance ligand 2; **AMOTL1**, Angiomotin Like 1; **FNDC1**, Fibronectin type III domain containing 1; **CDH11**, Cadherin 11; **DDR2**, Discoidin domain receptor tyrosine kinase 2; **CTHRC1**, Collagen triple helix repeat containing 1; **AKAP12**, A-kinase anchoring protein 12; **SFRP2**, Secreted frizzled related protein 2; **PTGR2**, Prostaglandin reductase 2.

To enhance our comprehension of the biological relevance of the 12 DEGs in the context of colorectal cancer, the gene signatures were subjected to IPA software library was used to generate a schematic network of gene signatures in different signaling pathways, elucidating their effect on the response of colorectal cancer patients to FOLFIRI drug (Figure 8). CD36 mediates signaling via either APP or JNK, thereby contributing to the activation of the inflammation and/or induction of apoptosis. SLIT2 mediates various signaling cascades including ROBO1/Beta-catenin or ROBO1/srGAPs/CDC43/P21-CIP, HGF/HGF/MET/GRB2/Ras/MAPK, SDF1/PI3K/CDC42/P21-CIP or SDF1/CXCR4/MMP9, and Netrin-1/DCC/Caspase3/YAP/TAZ/TEAD/AR contributing to cell adhesion, cell cycle arrest, cell invasion, and/or apoptosis. Activated by FAT4, AMOTL1 is linked to YAP/TAZ/TEAD, triggering apoptosis. FNDC1 and CDH11 activate AR, leading to the promotion of cell proliferation. AKAP12, activated by integrins, is linked to either RAF/MEK/CyclinD or JNK/AP-1, leading to the activation of HIF-1/VEGF which, in turn, trigger angiogenesis and cell proliferation. SFRP2 is associated with the complex Wnt/LRP5/6. This complex mediates signaling via DVL/RAC1/JNK/AP-1/HIF-1/VEGF leading to angiogenesis. Both DDR2 and CTHRC1 are activated by TRIM67. Finally, PTGR2 is indirectly activated by either of these signals LPS/IL-1β/IL-6/TNF-α/IL-18. These signals mediates signaling cascade PLA2/Arachidonic Acid/COX/15k-PGE2/Keap1/Nrf2. The activation of PTGR2 transforms 15k-PGE2 to 13,14 dihydro 15k-PGE2 leading to the degradation of Nrf2 nd the activation of pro-inflammatory cytokines.

Additional analysis of the changes in the expression levels of shed some insights into the mechanisms involved in the response to FOLFIRI in colorectal cancer.

CD36—Cluster of differentiation 36 (CD36) activates MAPK which activates JNK that can lead to apoptosis (Silverstein and Febbraio, 2009; Feng et al., 2023). It is though to be important in many types of cancer and is high expression of CD36 is correlated with cancer drug resistance, including irinotecan (Jiang et al., 2019; Drury et al., 2020; Gyamfi et al., 2021; Feng et al., 2023). The responders to FOLFIRI show reduced expression of CD36 compared to the non-responders in the study data.

SLIT2 - Slit guidance ligand 2 (SLIT2) has been observed to have tumor suppressing activity (Zhao et al., 2018). SLIT2 is the ligand of roundabout guidance receptor 1 (ROBO1). Together they play a role in cancer cell proliferation, apoptosis, migration and invasion, and angiogenesis (Zhao et al., 2018). Additionally, in colon cancer, SLIT2/ROBO1 has been shown to encourage tumor growth. On the other hand, SLIT2 has been shown to suppress β-catenin levels which are positively correlated with chemotherapy resistance (Ahirwar et al., 2021; Ahirwar et al., 2023). The responders show a reduced SLIT2 expression which should reduce the contributions of SLIT2 signaling. It is not clear how this contributes to FOLFIRI sensitivity.

AMOTL1—AMOTL1 encodes angiomotin1 which bind the protein YAP1 in the cytoplasm and protects it from degradation (Zhou et al., 2020). YAP1 has been observed to increase cancer drug resistance. Inhibition of the expression and activation of YAP1 is a major way utilized to overcome drug resistance (Liu et al., 2020). The reduced expression of AMOTL1 in FOLFIRI responders vs. non-responders suggests that there is less drug resistance conferred because YAP1 can be degraded more easily without AMOTL1.

FNDC1 - Fibronectin type III domain containing 1 (FNDC1) activates a G-protein signaling cascade that leads to the activation of PI3K/Akt/mToR signaling which leads to cancer growth and proliferation (Chen et al., 2022). Furthermore, FNDC1 overexpression improved cell survival during chemotherapy (5-FU). In addition, CRC tissues from non-responders were found to exhibit higher level activation of the signaling PI3K/Akt compared to responders (Chen et al., 2022). The reduction in FNDC1 expression seen in responders compared to non-responders suggests that there will be reduced chemotherapy resistance.

CDH11 - Cadherin-11 (CDH11) has been associated with aggressive cancer (Yang et al., 2021). CDH11 mediates cell-to-cell and cell-to-matrix adhesion. Upregulated CDH11 has been linked to increased metastases through the activation of NF-κB (Wang et al., 2020). Reduced CDH11 expression observed in the responders to FOLFIRI compared to the non-responders might be correlated with reduced metastases and better response.

DDR2 - Discoidin Domain Receptor 2 (DDR2) is a tyrosine kinase receptor that binds to collagen (Lafitte et al., 2020). Activation of DDR2 by collagen activates growth and proliferation through the Ras/Rac/MEK/ERK and PI3K/Akt/mTOR pathways (Lafitte et al., 2020). Reduction in DDR2 through knock-out in mice showed decreased cell proliferation. Decreased DDR2 in colorectal cancer showed reduced metastasis (Lafitte et al., 2020). The reduced DDR2 expression seen in FOLFIRI responders might lead to treatment success due to reduced metastases.

CTHRC1 - Collagen triple helix repeat containing 1 (CTHRC1) is involved in tissue repair and is highly expressed in various malignant tumors including colorectal cancer (Liu et al., 2023). CTHRC1 activates Wnt signaling as well as the PI3K/ERK pathway (Liu et al., 2023). Activation of Wnt is associated with chemotherapy resistance. Activation of PI3K and ERK is associated with cell growth and proliferation (Liu et al., 2023). *In vivo* analysis showed that knocking down of CTHRC1 from CRC cell line inhibits the formation of tumor (Liu et al., 2023). The reduced expression of CTHRC1 in FOLFIRI responders would make these patients’ cancer more sensitive to chemotherapy.

AKAP12 - A-kinase (PRKA) anchor protein 12 (AKAP12) anchors protein kinase A and protein kinase C to the plasma membrane (He et al., 2018; Liang et al., 2022). AKAP12 was found to be downregulated in almost 50% of CRC tissues as compared with their matched non-tumor tissues (He et al., 2018). AKAP12 has been observed to suppress Src-induced oncogenic proliferation, invasiveness, and cell death through its interactions with SRC (He et al., 2018). The reduced AKAP12 expression seen in FOLFIRI responders vs. non-responders might be because these cancers do not have cell death inhibited.

SRFP2 - SRFP2 works with the Wnt/β-catenin signaling pathway to promote cell homeostasis and contribute to chemoresistance (Sun et al., 2016). Wnt/β-catenin signaling promotes drug resistance through sensitization of the ABCB1 transporter (Zhu et al., 2021). It has been reported that overexpression of SFRP2 promotes the expression of YAP1 and the overexpression of YAP1 and SFRP2 promote the expression of β-catenin in CRC cells (Bai et al., 2021). The FOLFIRI responders showed reduced expression compared to non-responders to FOLFOX (logFC = −3.12). The lower SFRP2 likely abrogates chemoresistance signaling.

PTGR2 - Prostaglandin reductase 2 (PTGR2) catalyzes the NADPH-dependent reduction of 15-keto-PGE2 as a part of lipid metabolism (Chang et al., 2016). Gene silencing of PTGR2 suppressed pancreatic cancer cell growth and induced cancer cell death through increased 15-keto-PGE2 and ROS levels (Chang et al., 2016). PTGR2-knockdown gastric cancer cells rendered them more sensitive to cisplatin and 5-FU compared with the PTGR2-overexpressing cells (Gan et al., 2019). Lipid uptake, storage, and metabolism is upregulated in cancer to meet the increased energy demands (Cheng et al., 2022), The higher PTGR2 expression in FOLFIRI responders might align with the increase metabolism in the colorectal cancer cells making them more susceptible to FOLFIRI.

COL1A2—COL1A2 encodes for type I collagen. Type I collagen binds receptors on the surface of tumor cells that result in tumor cell proliferation and metastasis (Shi et al., 2022). This occurs by the activation of the Ras/Raf/MEK/ERK and PI3K/Akt/mTOR pathways. Type I collagen also regulates the efficacy of chemotherapy (Shi et al., 2022). This concurs with the observation that reduced COL1A2 expression in cells responding to FOLFIRI. COL3A1—COL3A1 encodes for type III collagen (Wang et al., 2022). High levels of COL3A1 is associated with poor prognosis of the cancer patient because it promotes cell viability and inhibits apoptosis (Wang et al., 2022). This occurs by the activation of the Ras/Raf/MEK/ERK and PI3K/Akt/mTOR pathways. The reduction of COL3A1 expression in responders is consistent with these observations.

### 4.5 Study limitations

This study has some limitations. For instance, FOLFOX and FOLFIRI treatment response prediction were performed in small datasets because the datasets were divided into subgroups to separate primary from metastatic CRC samples. Despite these limitations, it appears that machine learning models can predict the drug response of colorectal cancer patients on this specific data set. Further optimization and validation on larger datasets is required to determine if this approach is clinically applicable.

The use of feature selection in this study has improved the accuracy, sensitivity, and specificity of the random forest model for predicting drug efficacy. Other studies have also seen and improved model performance after feature selection (Sharma and Dey, 2021). In some of the studies the accuracy, sensitivity, and specificity during training and validation was 1. This does not mean that the model will be 100% accurate on a separate test data set. This can be seen clearly in Table 5 where the model performance is lower on the independent test set compared to the training data. Unfortunately, in some of the cases, an independent test data set was not available, and the existing data set was small so creating a test data set was not practical.

## 5 Conclusion

In conclusion, the current study identified gene signatures that could predict for the response to 5-FU based chemotherapy in patients with colorectal cancer with high accuracy. The application of the machine learning models to the data sets obtained from GEO suggested that 28.6% of patients who failed the treatment therapy they received would benefit from the alternative treatment. Application of this machine learning approach predicts strategies that might improve drug treatment outcomes for patients with CRC and other cancers. After additional clinical validation, this approach has significant potential for integration into clinical practice.

Analysis of the gene signatures gives the following insights into the important mechanism for FOLFOX sensitivity in both early-stage and metastatic colorectal cancer. The responders seem to have genes that encourage fast growth and proliferation through the MAPK/ERK/MEK and cAMP/PKA signaling pathways and have increased metabolism making them more sensitive to chemotherapy. Cell death through apoptosis or anoikis is not inhibited responders compered to non-responders through pathways such as MAPK/JNK/Jun and cell-death due extracellular matrix cell contact disruption. Furthermore, chemoresistance brought about by Wnt/β-catenin signaling and its role in chemoresistance through ABCB1 transporter expression. Finally, the tumor mechanisms for immune system evasion or causing inflammation seem to be inhibited by the gene expression changes.

Analysis of the gene signatures gives the following insights into the important mechanism for FOLFIRI sensitivity in both colorectal cancers. The gene expression changes result fast growth and proliferation (Ras/Raf/MEK/ERK and PI3K/Akt/mTOR) that is accompanies by increased metabolism. This makes the cancers more susceptible to chemotherapy agents such as FOLFIRI. Cell death through apoptosis or anoikis is less inhibited in the responders than non-responders making them more sensitive to death resulting from chemotherapy. Also, the suppression of the Wnt/β-catenin signaling in responders results in less chemoresistance by reducing ABCB1 transporter expression which exports chemotherapy agents.

## Data Availability

The datasets presented in this study can be found in online repositories. The names of the repository/repositories and accession number(s) can be found in the article/Supplementary Material.

## Supplementary material

SUPPLEMENTARY FIGURE S1 Protein-protein interaction networks. The network derived from IMEx interactome database (using NetworkAnalyst web-based visual analytics platform) shows interactions among the gene signatures that are associated to CRC patients who received FOLFOX drug. Genes in red represent the identified genes from the feature selection methods.

SUPPLEMENTARY FIGURE S2 Protein-protein interaction networks. The network derived from IMEx interactome database (using NetworkAnalyst web-based visual analytics platform) shows interactions among the gene signatures that are associated to early-stage CRC patients who received FOLFOX drug. Genes in red represent the identified genes from the feature selection methods.

SUPPLEMENTARY FIGURE S3 Protein-protein interaction networks. The network derived from IMEx interactome database (using NetworkAnalyst web-based visual analytics platform) shows interactions among the gene signatures that are associated to metastatic CRC patients who received FOLFOX drug. Genes in red represent the identified genes from the feature selection methods.

SUPPLEMENTARY FIGURE S4 Protein-protein interaction networks. The network derived from IMEx interactome database (using NetworkAnalyst web-based visual analytics platform) shows interactions among the gene signatures that are associated to CRC patients who received FOLFIRI drug. Genes in red represent the identified genes from the feature selection methods.
