# Supplementary figures and images for: High-accuracy prediction of colorectal cancer chemotherapy efficacy using machine learning applied to gene expression data
Source: Front Physiol. 2024 Jan 18;14:1272206. doi: 10.3389/fphys.2023.1272206 (PMC10830836; doi:10.3389/fphys.2023.1272206)

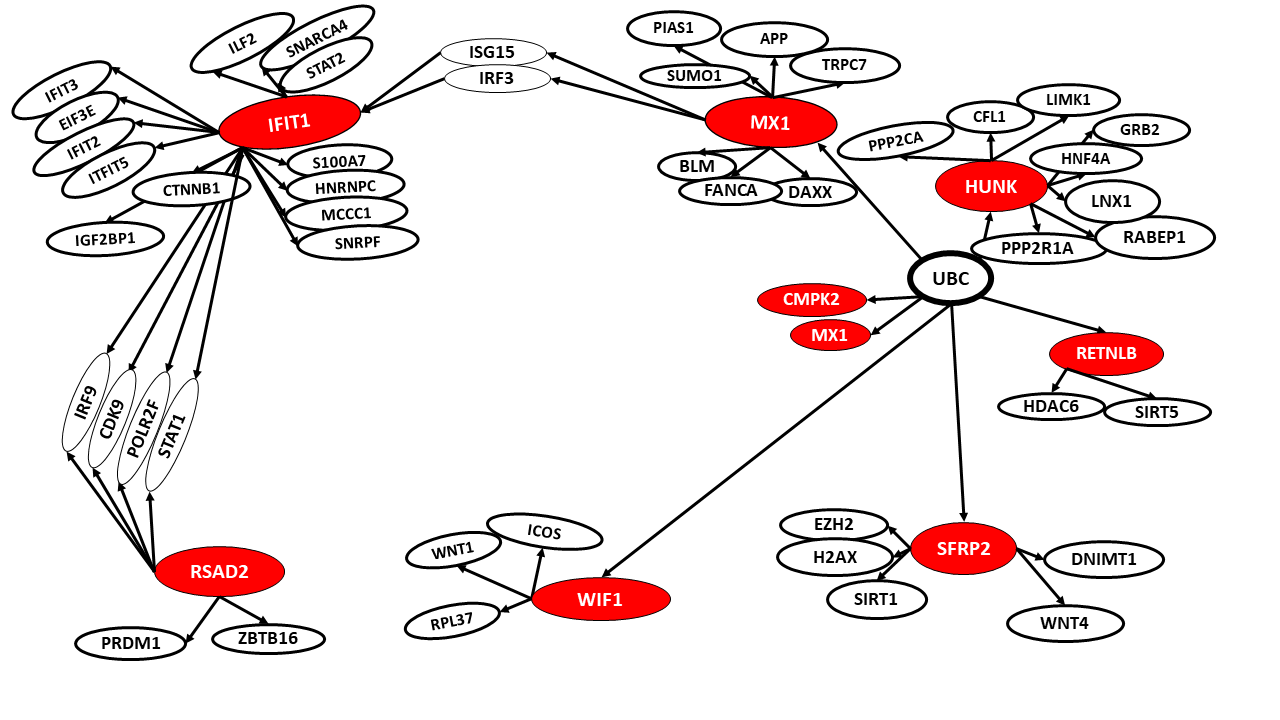

Supplement: Supplementary file 1 [file Image3.TIF]

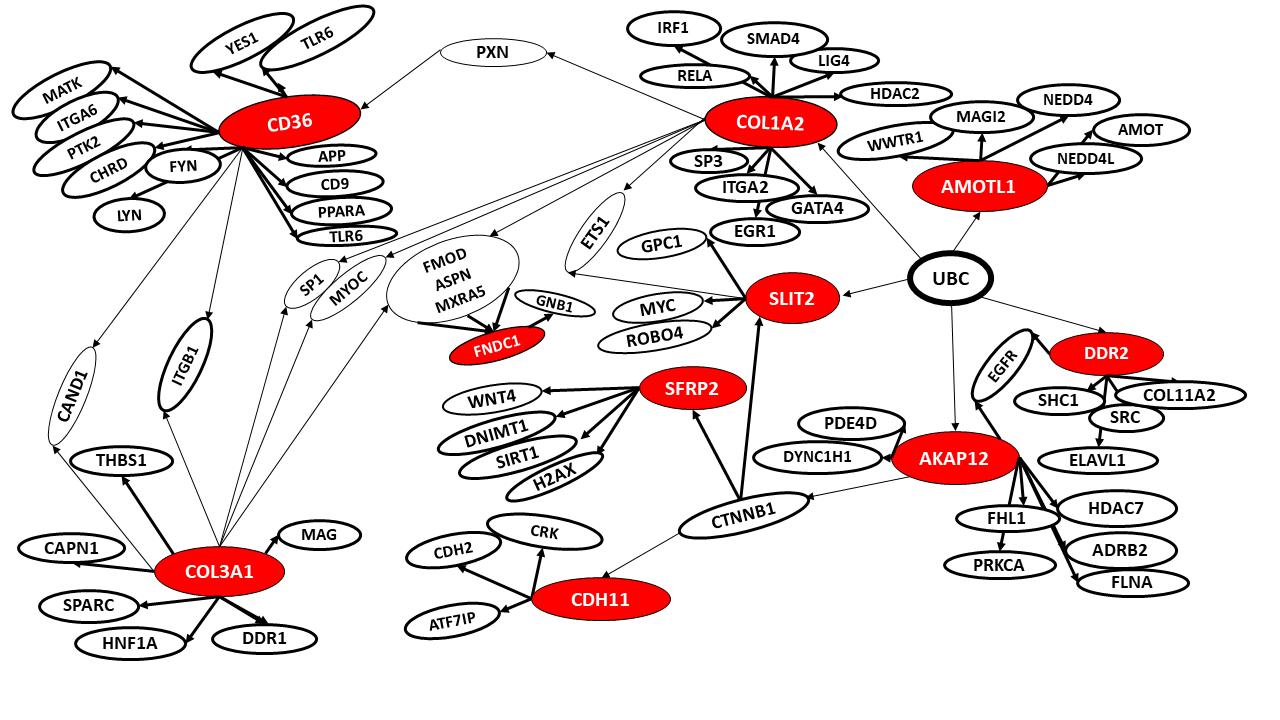

Supplement: Supplementary file 2 [file Image4.TIF]

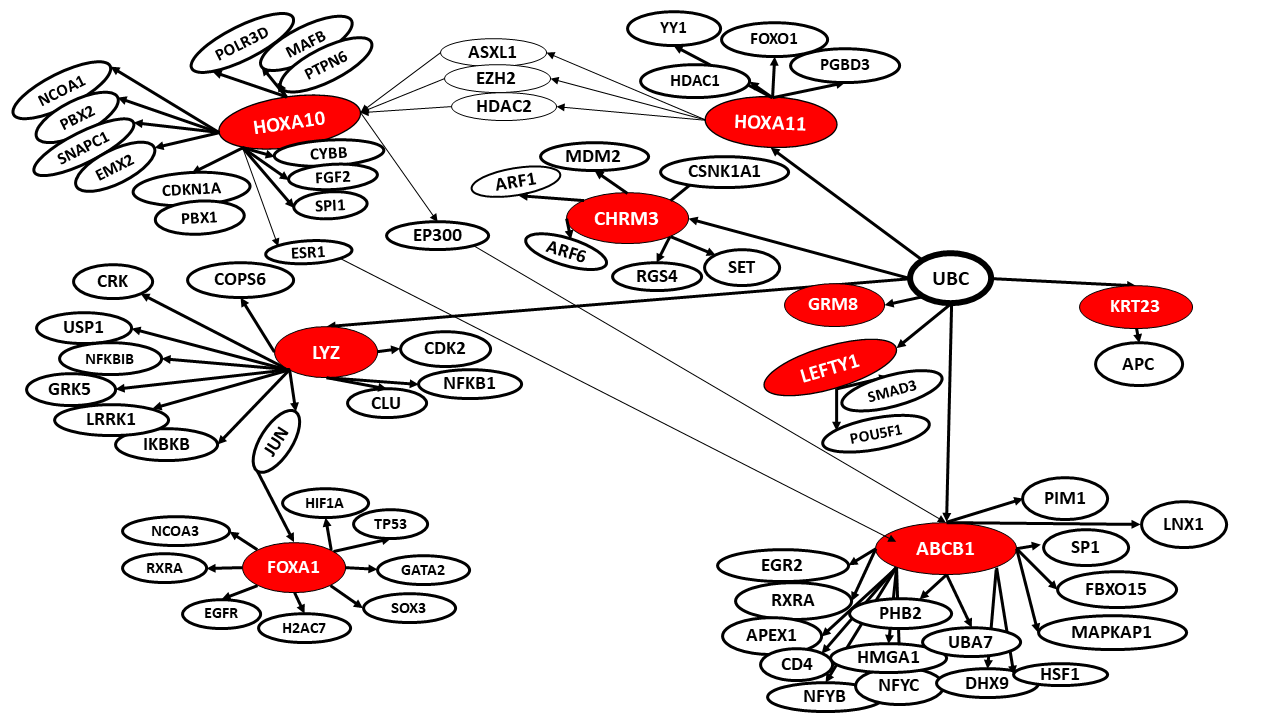

Supplement: Supplementary file 3 [file Image2.TIF]

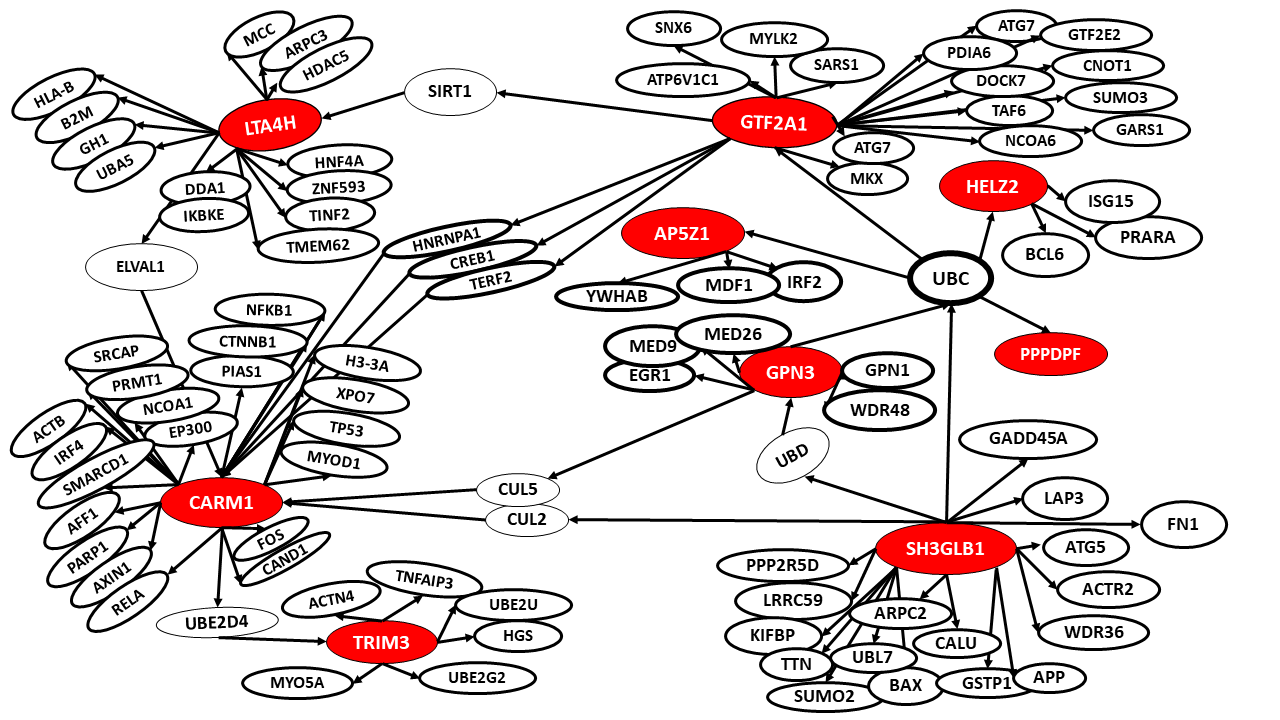

Supplement: Supplementary file 4 [file Image1.TIF]
